# *Cucumis sativus* L. Seeds Ameliorate Muscular Spasm-Induced Gastrointestinal and Respiratory Disorders by Simultaneously Inhibiting Calcium Mediated Signaling Pathway

**DOI:** 10.3390/ph14111197

**Published:** 2021-11-22

**Authors:** Muqeet Wahid, Fatima Saqib, Hanadi Talal Ahmedah, Claudia Mihaela Gavris, Vincenzo De Feo, Mircea Hogea, Marius Moga, Radu Chicea

**Affiliations:** 1 Department of Pharmacology, Faculty of Pharmacy, Bahauddin Zakariya University, Multan 60800, Pakistan; muqeetsoomro@msn.com (M.W.); fatima.saqib@bzu.edu.pk (F.S.); 2 Department of Medical Laboratory Technology, Faculty of Applied Medical Sciences, King Abdulaziz University, Rabigh 25732, Saudi Arabia; hehmedouh@kau.edu.sa; 3 Faculty of Medicine, Transilvania University of Brasov, 500036 Brasov, Romania; mircea87@gmail.com (M.H.); moga.og@gmail.com (M.M.); 4 Department of Pharmacy, Salerno University, Fisciano, 84084 Salerno, Italy; 5 Faculty of Medicine, Lucian Blaga University, 550024 Sibiu, Romania; radu.chicea@ulbsibiu.ro

**Keywords:** asthma, antidiarrheal, bronchodilator, *Cucumis sativus*, cucumber, HPLC, LC-ESI-MS/MS

## Abstract

*Cucumis sativus* L. is globally cultivated as an edible vegetable. Besides its nutritional benefits, it is used in traditional medicines against various ailments. The current study was designed to elucidate the multi-target mechanisms of a *C. sativus* seeds extract against asthma and diarrhea using network pharmacology along with a molecular docking approach. Furthermore, in-vitro and in-vivo experiments were conducted to verify the mechanistic insight of in silico studies. LC-ESI-MS/MS was performed to identify the bioactive compounds in the extract; later, some compounds were quantified by HPLC. *C. sativus* seed. EtOH has kaempferol in higher concentration 783.02 µg/g, followed by quercetin (693.83 µg/g) and luteolin (617.17 µg/g). In silico studies showed that bioactive compounds interfered with asthma and diarrhea-associated target genes, which are members of calcium-mediated signaling to exert a calcium channel blocker activity. The seeds extract exerted a concentration-dependent spasmolytic response on isolated jejunum, trachea, and urinary bladder preparations and caused relaxation of spastic contraction of K^+^ (80 mM) with suppressed calcium concentration-response curves at dose 0.3 and 1 mg/mL. It also showed antiperistalsis, antidiarrheal and antisecretory activity in animal models. Thus, *C. sativus* seeds have therapeutic effects by regulating the contractile response through a calcium-mediated signaling pathway.

## 1. Introduction

*Cucumis sativus* L., the cucumber, belongs to the Cucurbitaceae family and is widely cultivated as an edible vegetable, especially in tropical areas of Africa, Asia, and South America [1,2] and is commonly used as a green appetizer and eaten with a main meal due to the presence of vitamins, proteins, carbohydrates, and fatty acids [1]. Herbal practitioners of Pakistan and India use its seeds to cure antitussive [3,4] and urinary problems [5,6]. Its fruits can aid in constipation and indigestion [7] due to their purgative [8], stomachic [9], antacid, and carminative properties [10]. Fruit is also used to cure burning sensations, burns, and open sores [11]. It has antioxidant, anti-ulcer [11], cytotoxic [12], inflammatory, antihypertensive [13], and analgesic properties [14]. *C. sativus* seeds are used against hyperlipidemia [15], diabetes mellitus [16], intermittent fevers, analgesic, burning sensations, and inflammation [17,18]. Gill et al. [19] reported that *C. sativus* seeds had anti-ulcer and anti-depression properties. In Unani medicine systems, *C. sativus* seeds are used in the following unani compound formulation *Jawarish zarooni sada* (manage urinary bladder and kidney functions) [5], *Sharbat-e-bazoori mutadil* (improve liver and bladder problems) [20], *Banadiq ul buzoor* (renal disorders) [21], *Qurs zarishk* (hepatoprotective) [22], *Qurs sartan kafoori* (to treat chronic bronchitis, tuberculosis, and cough) [3], *Laooq badam* (dry cough and tuberculosis), *Qurs kafoor* (to treat diabetes), and *Laboob barid* [23,24].

*C. sativus* seed and fruit contain volatile oil, fixed oil, flavones, steroids, flavonoids, tannins, phytosterols, saponin, cucurbitacins, and alkaloids [6,12,25]. The medicinal plants with similar phytochemical profiles exhibited a therapeutic potential for treating gastrointestinal and respiratory ailments [26]. These secondary metabolites have been reported for spasmolytic activity through calcium influx inhibition, potassium channel activation, and muscarinic receptor antagonism [26].

Hence, the primary purposes of this study were (1) to characterize the bioactive compounds of a hydroethanolic *C. sativus* seeds extract; (2) to assess its possible antidiarrheal and bronchodilator properties; and (3) to explore the pharmacodynamic mechanisms in vitro, in vivo, and in silico models.

## 2. Results

### 2.1. Identification of Bioactive Compounds by LC-ESI-MS/MS Analysis

**Figure 1:**
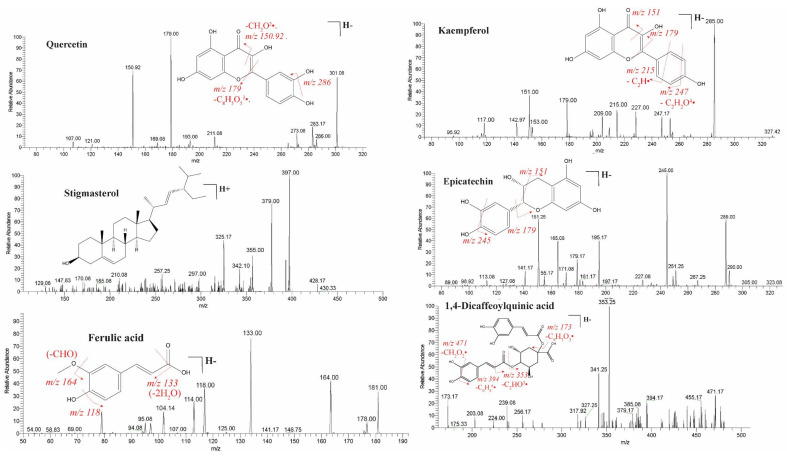

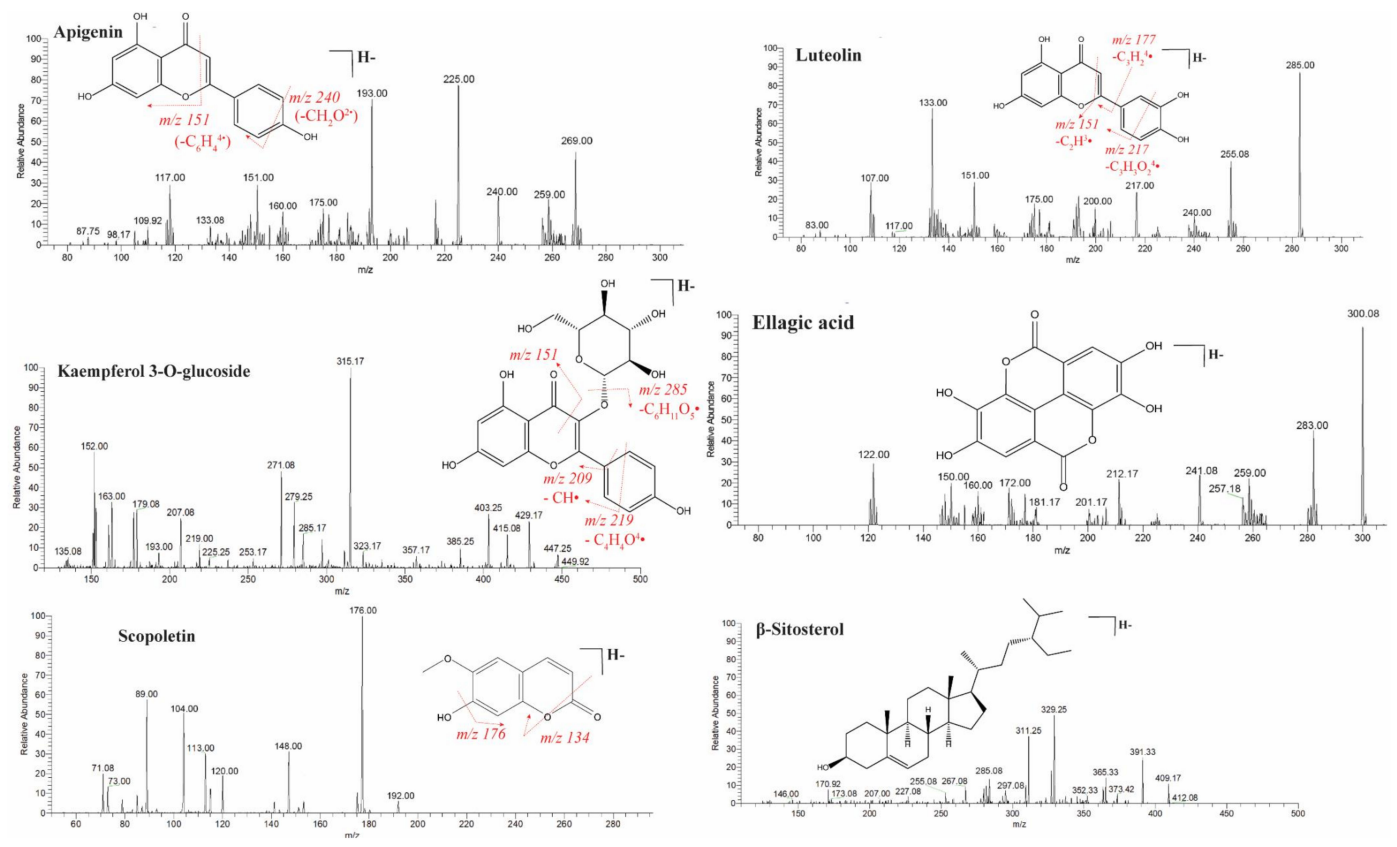
ESI-MS/MS spectra in negative and positive mode for tentative compounds of a hydroethanolic extract of *C. sativus* seeds. The tentative compounds are quercetin, kaempferol, stigmasterol, epicatechin, ferulic acid, 1,4-dicaffeoylquinic acid, apigenin, luteolin, kaempferol-3-*O*-glucoside, ellagic acid, scopoletin, and β-sitosterol.

LC-ESI-MS/MS analysis indicated the presence of 30 components. The major compounds identified were stigmasterol, β–sitosterol, scopoletin, 1,4-dicaffeoylquinic acid, luteolin, kaempferol, ferulic acid, epicatechin, ellagic acid, kaempferol-3-*O*-glucoside, quercetin, and apigenin (Figure 1 and Table 1). These bioactive compounds are collectively responsible for the pharmacological activities of *C. sativus*.

### 2.2. Optimization of HPLC Conditions and Method Validation

**Figure 2:**
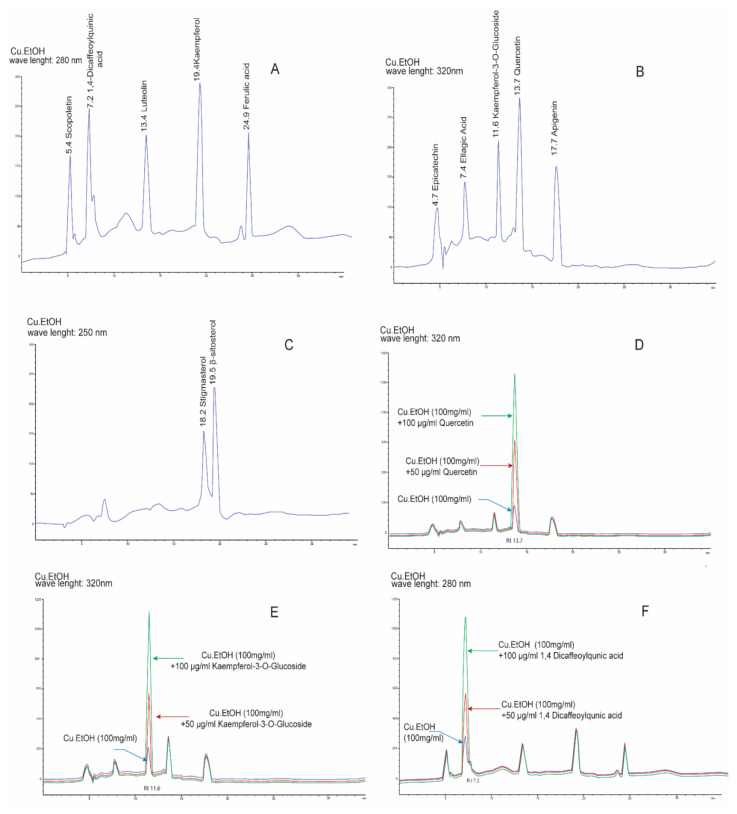

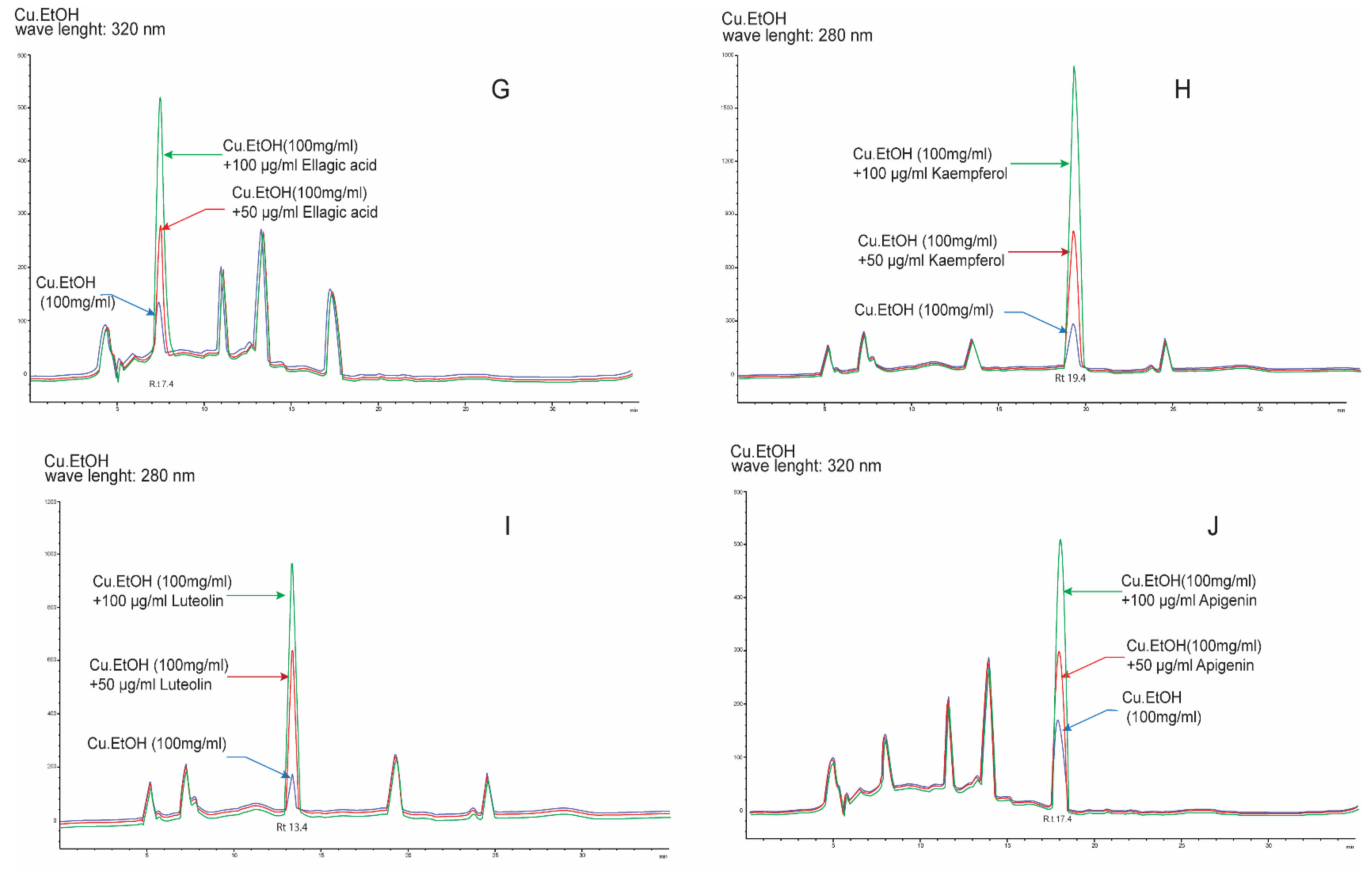
HPLC DAD-UV/Vis chromatograms of a hydroethanolic extract of *C. sativus* seeds at different wavelengths and standard addition validation chromatograms of bioactive compounds. HPLC chromatogram of (**A**) scopoletin, 1,4-dicaffeoylquinic acid, luteolin, kaempferol, and ferulic acid wavelength 280 nm; (**B**) epicatechin, ellagic acid, kaempferol-3-*O*-glucoside, quercetin, and apigenin at wavelength 320 nm; (**C**) Stigmasterol and β–sitosterol at wavelength 250 nm. The standard addition chromatograms of (**D**) quercetin; (**E**) kaempferol-3-*O*-Glucoside; (**F**) 1,4 dicaffeoylqunic acid; (**G**) ellagic acid; (**H**) kaempferol; (**I**) luteolin (**J**) apigenin.

HPLC conditions were optimized to obtain maximum separation and visible peaks. A binary mobile phase system consisting of solvent A (0.1% trifluoroacetic with methanol) and solvent B (0.1% trifluoroacetic with acetonitrile) at 0.8 mL/min showed the maximum separation with a total run time of 40 min. Retention times of separation peaks were compared with those of external standards under the same chromatographic conditions. The peak of stigmasterol and β–sitosterol was visible at 250 nm, scopoletin, 1,4-dicaffeoylquinic acid, luteolin, kaempferol, and ferulic acid had visible peaks at 280 nm; epicatechin, ellagic acid, kaempferol-3-*O*-glucoside, quercetin, and apigenin showed a better response at 320 nm (Figure 2, Table 2).

The calibration curves of dilutions of external standards were used for linearity validation. The linearity range of standards curves of stigmasterol, β–sitosterol, scopoletin, 1,4-dicaffeoylquinic acid, luteolin, kaempferol, ferulic acid, epicatechin, ellagic acid, kaempferol-3-O-glucoside, quercetin, and apigenin was found between 7.81 and 500 µg/mL with a significant regression coefficient (r^2^) 0.9991–0.9999. LOD and LOQ were found between 0.20–0.63 and 0.61–1.93 µg/mL, respectively, as shown in Table 2. The instrumental precision and repeatability were validated by inter-day (*n* = 3) and intra-day for three consecutive days (*n* = 9) analysis of external standards. In precision validation (Table 2 and Table S1), the results showed the inter-day and intra-day had %RSD between 0.44–1.75 and 0.40–1.67%. In accuracy validation, the extract was spiked with different concentrations of external standards (50, 100, 150 µg/mL) to calculate the recovery percentage. The results in recovery percentage (Table 2 and Table S2) showed that the mean value ranged from 98.58 ± 0.46 to 100.00 ± 0.82 with %RSD 0.06–1.51, which depicts the good accuracy of this method (Table 2 and Table S2).

#### Quantification Analysis of Bioactive Compounds by HPLC

The compounds identified by LC ESI-MS/MS were quantified based on standard calibration curves of external standard dilutions. Kaempferol resulted in the higher concentration 783.02 µg/g, followed by quercetin (693.83 µg/g), luteolin (617.17 µg/g), apigenin (578.93 µg/g), and ellagic acid (542.71 µg/g) (Table 2). Kaempferol-3-*O*-glucoside, 1,4-dicaffeoylquinic acid, epicatechin, ferulic acid, β-sitosterol, stigmasterol, and scopoletin, were quantified 457.81, 452.18, 370.45, 355.35, 317.04, 243.66, 217.4 µg/g, respectively, based on standard dilutions calibration curves.

### 2.3. In Silico Studies

#### 2.3.1. ADMET and Drug-Likeness

The previously identified compounds were studied in Qikprop, SWISS ADME, and pkCSM for ADMET and drug-likeness (DL) (Table 3) to predict the following parameters: octanol/water partition coefficient, aqueous solubility; GI absorption; blockage of HERG K^+^ channels; gut blood barrier permeability, brain–blood barrier; skin permeability; human serum albumin binding; the volume of distribution; CNS permeability; bioavailability score; *P* glycoprotein transportation and contraindication with other drugs, primary metabolites; CYP450 enzymes metabolism; total clearance, and toxicity studied in AMES toxicity; oral rat acute toxicity (LD_50_); oral rat chronic toxicity (LOAEL); hepatotoxicity and skin sensitization.

All compounds were retrieved with noteworthy profiles. Therefore, ADMET analysis helped in the selection of ligands owning pivotal pharmacokinetic properties within acceptable ranges. All compounds had favorable pharmacokinetic parameters with no significant adverse effects, with potential medicinal use.

It is challenging for bioactive compounds of natural origin to fit within desired ranges of molecular weight and lipophilicity partition coefficient (log Po/w), a lipophilicity parameter. All isolated compounds had a molecular weight less than 500 Dalton, except 1,4-dicaffeoylquinic acid, and passed the lipophilicity partition coefficient except hydrophobic phytosterols, i.e., β-sitosterol and stigmasterol. Again, the aqueous solubility of most compounds lies within the permissible range except hydrophobic phytosterols. Most compounds showed poor GI absorption. This limitation can be tackled during formulation development. All compounds except 1,4-dicaffeoylquinic acid and kaempferol-3-*O*-glucoside showed moderate or good permeability for CNS and blood–brain barrier. Ellagic acid and quercetin possessed a slight permeability for CNS and blood–brain barrier. Most compounds fit in the permissible range of blockage of HERG K^+^ channels, human serum albumin binding (except β-sitosterol and stigmasterol), bioavailability score, and volume of distribution. All these parameters permit a plausible distribution of ligands in the body. All bioactive compounds screened for ADMET passed the metabolic, renal excretion, and toxicity parameters.

Lipinski and other parameters were studied for drug-likeness of compounds, but most compounds could not pass these drug-likeness filters sufficiently. Lipinski’s rule of five and other parameters are not considered rigid standards because FDA approved most natural products and semisynthetic derivatives that cannot fit with these parameters [27,28].

#### 2.3.2. Network Pharmacology Analysis

*Potential targets Screening:* The potential target profile of each compound was retrieved from Swiss target prediction, TCMSP, and Drugbank datasets with a cut-off probability score >0.5. A total of 269 target genes were gathered and retained after removing the duplicates. The gastrointestinal and respiratory disease-associated target genes were obtained for keywords “asthma”; “coughing”; “wheezing”; “diarrhea”; “constipation”; and “irritable bowel syndrome” from the Gene card, DisGeNET, Pubmed, and OMIM databases. The target genes of gastrointestinal and respiratory disorders were sorted separately, corrected with Uniprot, and subjected to the VarElect tool to validate and score the genes. The top 200 genes were kept from gastrointestinal and respiratory disorder gene datasets [29,30]. The disease target genes and 269 compound target genes were intersected in the Venn diagram, revealing 57 intersected potential targets for gastrointestinal and respiratory disorders (Figure S1). These targets (Table S3) were retained for network construction, gene ontology (GO), and KEGG pathway analysis.

**Figure 3:**
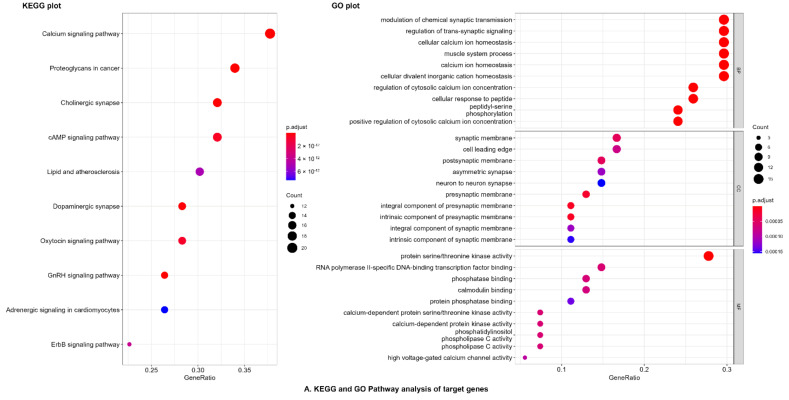

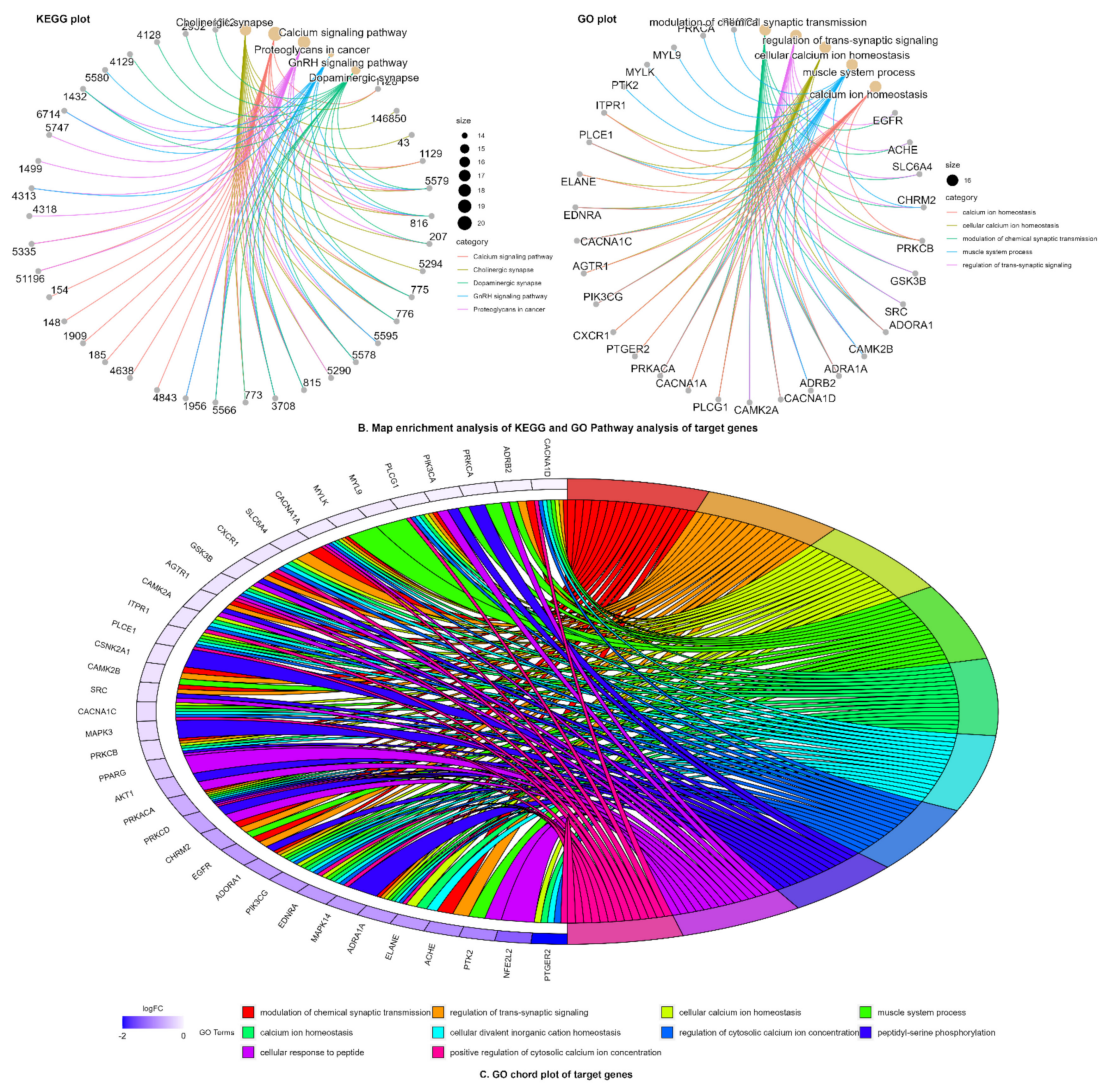
KEGG and GO biological analysis of bioactive compounds of *C. sativus* seeds EtOH extract. (**A**) Dot plot (**B**) Map enrichment and (**C**) Chord plot of KEGG and GO biological analysis.

KEGG and GO analysis: The target genes were subjected to R studio packages “BiocManager,” “Clusterprofiler,” and “org.Hs.eg.db” for gene ontology (GO) and KEGG pathway analysis for underlying mechanism(s) of gastrointestinal and respiratory disorders. The targets of active compounds significantly enriched in several GO terms with prominence in the modulation of chemical synaptic transmission; cellular calcium ion homeostasis; muscle system process; regulation of cytosolic calcium ion concentration; ERBB signaling pathway, and muscle contraction in targets gene of both diseases (Table S4, Figure 3).

**Figure 4:**
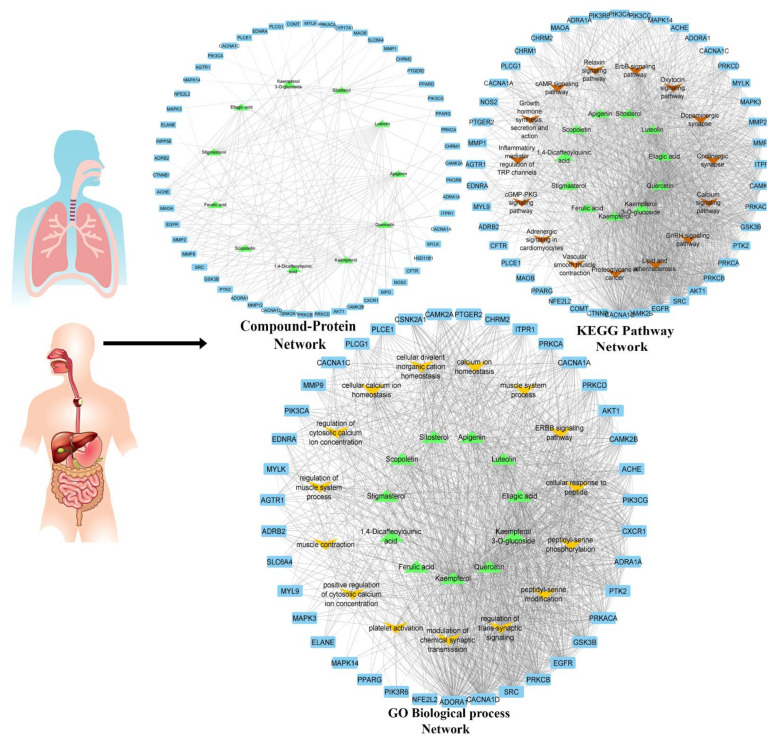
Network interaction analysis of bioactive compounds of *C. sativus* seeds with target disease proteins (C-T-D), compound target pathway (C-T-P) network interaction KEGG and GO biological process pathways.

KEGG signaling pathways involved in gastrointestinal and respiratory diseases are shown in Figure 4. The major KEGG signaling pathways regulated by active compounds of *C. sativus* were cholinergic synapse; calcium signaling pathway; proteoglycans in cancer; cAMP signaling pathway; and adrenergic signaling in cardiomyocytes (Figure 3, Table S5).

*Network Construction:* All retained key targets were subjected to the STRING plugin of Cytoscape for protein–protein interaction (PPI). The target genes had 54 nodes with 305 edges (Figure S2). A compound target disease (C-T-D) network was constructed in Cytoscape between bioactive compounds of *C. sativus* and target genes, which had 65 nodes and 161 edges. The network analysis showed that kaempferol had more significant interaction for targets genes with degree 27, followed by quercetin (degree = 21), apigenin (degree = 17), luteolin (degree = 17), and apigenin (degree = 13) (Table S6). Then, a compound target pathway (C-T-P) network interaction was built for GO biological process terms (Table S7) and KEGG pathways (Table S8), genes, and compounds (Figure 4). Only kaempferol, kaempferol-3-O-glucoside and quercetin were prominent in network analysis.

**Figure 5:**
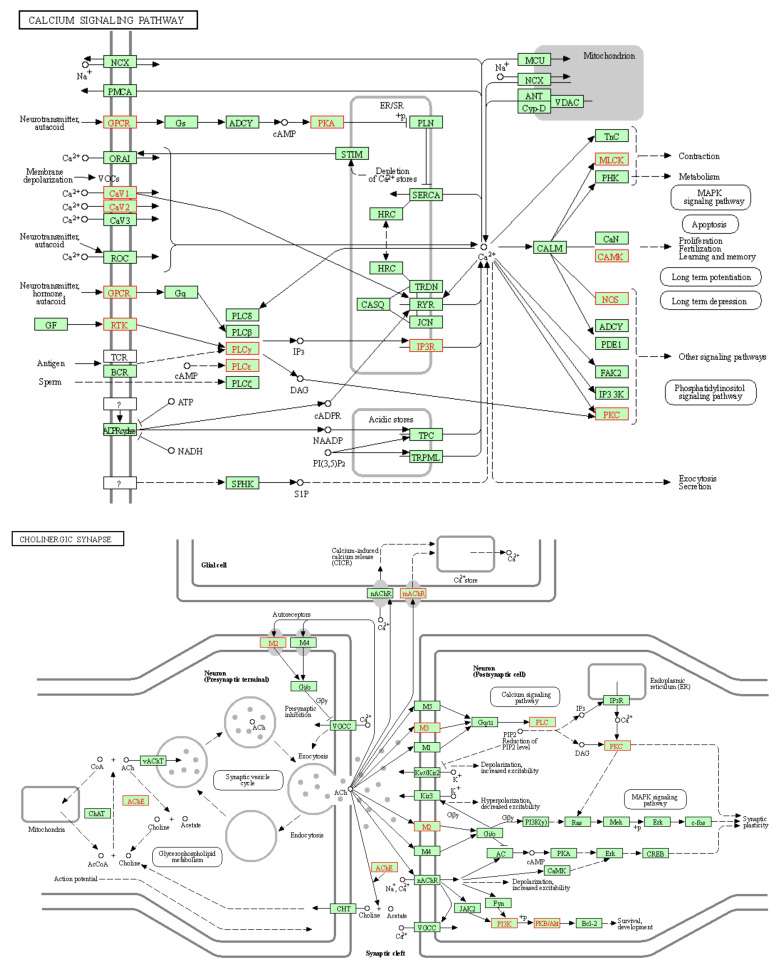
KEGG pathway for calcium mediates signaling and cholinergic synapse. The red boxes indicate the possible proteins targets of compounds.

The network analysis of these interactions showed that kaempferol-3-*O*-glucoside, luteolin, ellagic acid, kaempferol, and quercetin interfere with these target genes along other genes in calcium-mediated signaling and cholinergic synapse pathway (Figure 5) for possible antispamodic activity. These bioactive compounds and verapamil (standard calcium channel blocker (CCB)) were further investigated for molecular docking to predict the binding forces and stability of active compounds to target proteins.

#### 2.3.3. Molecular Docking

**Figure 6:**
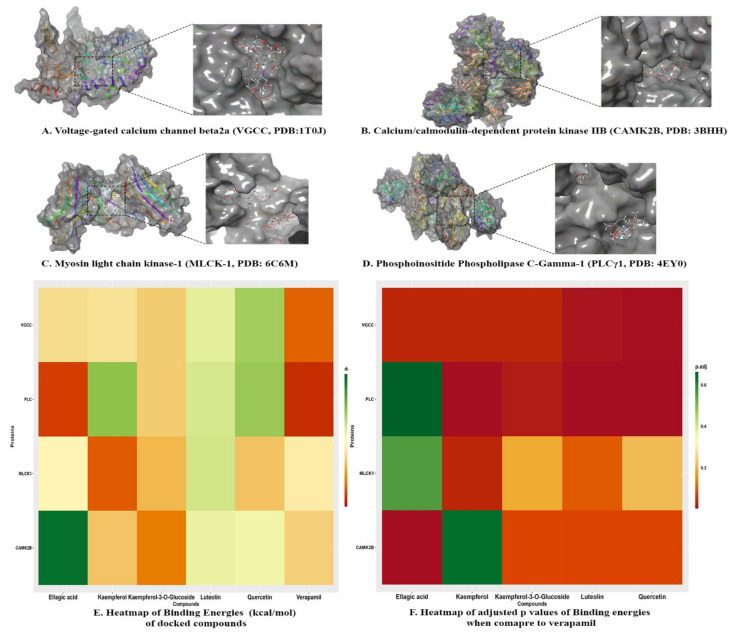
3D protein–ligand interaction between bioactive compounds (verapamil, kaempferol, quercetin, ellagic acid, luteolin and kaempferol-3-*O*-glucoside) and proteins; (**A**) voltage-gated calcium channel β2a (VGCC, PDB:1T0J); (**B**) calcium/calmodulin-dependent protein kinase IIB (CAMK2B, PDB: 3BHH); (**C**) myosin light chain kinase-1 (MLCK-1, PDB:6C6M); and (**D**) phosphoinositide phospholipase C-gamma-1 (PLCγ-1, PDB: 4EY0). (**E**) Heatmap of binding energies (kcal/mol), of bioactive compounds (verapamil, kaempferol, quercetin, ellagic acid, luteolin and kaempferol-3-*O*-glucoside) and proteins. (**F**) Heatmap of p-adjusted values of binding energies when compared to verapamil. (*p* < 0.05 vs. verapamil).

**Figure 7:**
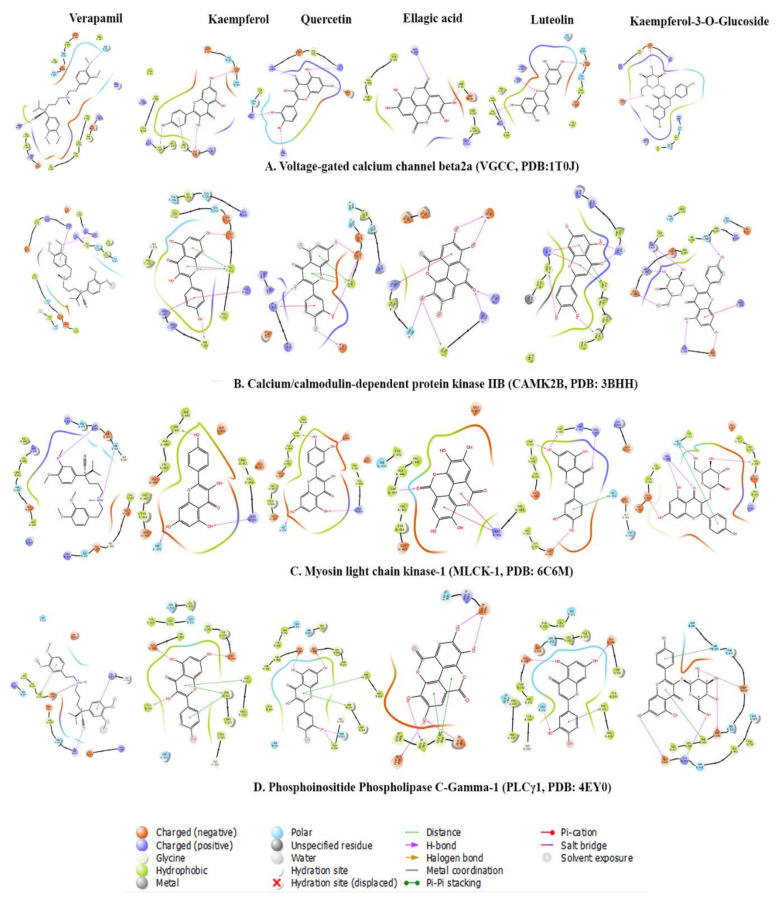
2D protein–ligand interaction between bioactive compounds (verapamil, kaempferol, quercetin, ellagic acid, luteolin and kaempferol-3-*O*-glucoside) and proteins; (**A**) voltage-gated calcium channel β2a (VGCC, PDB:1T0J); (**B**) calcium/calmodulin-dependent protein kinase IIB (CAMK2B, PDB: 3BHH); (**C**) myosin light chain kinase-1 (MLCK-1, PDB:6C6M); and (**D**) phosphoinositide phospholipase C-gamma-1 (PLCγ-1, PDB: 4EY0).

The docking calculations are beneficial to predict ligand impacts within the binding site of a target protein. The involvement of physical energies terms (i.e., solvation energy) with a suitable force field makes the docking calculation of compounds more acceptable with accuracy [31,32]. The bioactive compounds rutin, kaempferol, scopoletin, and quercetin were docked with target proteins; voltage-gated calcium channel β2a (VGCC, PDB:1T0J), calcium/calmodulin-dependent protein kinase IIB (CAMK2B, PDB: 3BHH), myosin light chain kinase-1 (MLCK-1, PDB:6C6M) and phosphoinositide phospholipase C-gamma-1 (PLCγ-1, PDB: 4EY0) were used to investigate plausible mechanistic pathway. These proteins are members of calcium-mediated signaling and cholinergic synapse pathways (Figure 5). Kaempferol-3-*O*-glucoside, kaempferol, and quercetin were predicted with the lowest binding energies and prominent bioactive compounds, with the contribution of van der Waals (ΔG_vdW_) and lipophilic interaction (ΔG_Lipo_) energies for target proteins. Kaempferol and quercetin also showed remarkable binding affinities towards target proteins, similar to verapamil (Table 4, Figure 6 and Figure 7).

*Voltage-gated calcium channel β2a*: Kaempferol-3-O-glucoside (∆G _Binding_: −36.36 kcal/mol) had dominant binding energies for voltage-gated calcium channels. It formed a conventional hydrogen bond with amino acids Lys254 (1.80Å), Arg424 (3.04Å), Asp251 (1.90Å), Asp319 (1.74Å) and Ile261 (2.88Å), carbon hydrogen bonds with amino acids Lys254 (3.03Å), Asp251 (2.91Å), and Asp319 (3.06Å). It had a hydrophobic π alkyl bond with amino acids Ile263 (4.40Å), Ile263 (4.75Å), and Arg265 (5.41Å). Luteolin (∆G _Binding_: −29.94 kcal/mol) formed with conventional hydrogen bond with amino acids Arg227 (2.41Å), Val109 (1.78Å), and Glu381 (1.73Å). It formed an electrostatic π anion bond with amino acids Asp384 (4.00Å) and Asp384 (3.53Å), and π−π T-shaped bond with amino acid Tyr402 (5.79Å). Ellagic acid (∆G _Binding_: −35.03 kcal/mol) formed a conventional hydrogen bond with amino acids Phe92 (1.95Å), Arg227 (1.98Å), and Tyr40 (2.89Å), carbon hydrogen bond with amino acids Asp91 (2.87Å), and Arg227 (2.99Å). It also had hydrophobic π- lone pair with amino acid Val109 (2.80Å), π−π Stacked bond with amino acid Tyr402 (5.40Å), and π -alkyl bond with amino acids with Lys110 (4.87Å), Val109 (5.29Å), Lys110 (5.49Å) and Ala405 (5.33Å). Kaempferol (∆G _Binding_: −34.67 kcal/mol) formed a conventional hydrogen bond with amino acids with Asp91 (2.73Å), Glu381 (1.81Å), and Val109 (1.75Å) and hydrophobic π−π T-shaped bond with amino acid Phe92 (5.30Å). Quercetin (∆G _Binding_ −26.09 kcal/mol) had conventional hydrogen bond with amino acids with Lys247 (2.09Å), Lys254 (1.93Å), Arg265 (1.96Å), Arg424 (2.14Å) and Asp319 (1.71Å) and hydrophobic π -alkyl bond with amino acids Lys247 (5.30Å) and Ile263 (5.49Å). Verapamil (∆G _Binding_: −42.52 kcal/mol), marketed calcium channel blocker, formed a conventional hydrogen bond with amino acid Arg227 (2.65Å) and carbon hydrogen bond with an amino acid with Tyr402 (2.72Å), Asp384 (2.54Å), Tyr402 (2.69Å), Glu111 (2.61Å), Ser330 (2.75Å), Pro336 (2.49Å), Glu381 (2.67Å) and Ser382 (2.43Å). It also formed an electrostatic attractive charge bond with amino acid Asp384 (4.51Å), π -cation bond with amino acid Arg227 (4.00Å), π-anion bond with amino acid Asp384 (3.66Å). Verapamil also formed hydrophobic alkyl with amino acids Ala335 (4.32Å), Ala405 (3.99Å), Lys110 (4.24Å), Pro326 (5.46Å), and Ile338 (4.46Å), π-alkyl bond with amino acids Phe92 (5.37Å), Lys110 (5.11Å) and Ala409 (4.44Å). The ranking order of ligands with VGCC based on docking score is given below: kaempferol-3-*O*-glucoside, luteolin, ellagic acid, kaempferol, quercetin, and verapamil.

*Calcium/calmodulin-dependent protein kinase IIB:* Kaempferol-3-O-Glucoside scored the lowest binding affinity for calcium/calmodulin-dependent protein kinase IIB (∆G _Binding_: −41.23 kcal/mol). It formed a conventional hydrogen bond with amino acids Arg187 (1.91Å), Asn256 (2.07Å), Lys138 (2.17Å), Lys227 (1.95Å), Lys227 (1.99Å), Glu189 (1.78Å), and Glu140 (1.86Å) and formed a carbon hydrogen bond with amino acids Glu189 (2.82Å), Glu189 (2.62Å). It also formed an electrostatic π -cation bond with amino acids Arg298 (4.27Å) and Arg298 (3.18Å). Kaempferol (∆G _Binding_: −36.84 kcal/mol) formed a conventional hydrogen bond with amino acid Trp215 (1.86Å), Glu217 (1.74Å), and Phe294 (2.13Å) and also formed π -donor hydrogen bond with amino acid Trp215 (2.44Å). It had electrostatic π -cation with amino acids Arg66 (3.87Å) and Arg298 (4.37Å), and hydrophobic interactions; π−π T-Shaped bond with amino acid Trp215 (4.32Å) and π -Alkyl bond with amino acids Arg297 (4.94Å) and Arg298 (4.83Å). Ellagic acid (∆G _Binding_: −18.31 kcal/mol) had a conventional hydrogen bond with amino acids Asn256 (2.69Å), Arg66 (2.59Å), Arg297 (2.09Å), Glu59 (2.02Å), Leu300 (2.02Å), Glu59 (1.89Å), and Leu300 (2.79Å), and carbon hydrogen bond with amino acid Arg297 (2.66Å). It also formed electrostatic π -cation bond with amino acids Lys259 (4.40Å) and Lys259 (3.50), electrostatic π -anion bond with amino acids Glu83 (3.77Å), Glu83 (3.94Å), and Glu83 (3.29). Quercetin (∆G _Binding_: −30.78 kcal/mol) formed a conventional hydrogen bond with amino acids Arg66 (2.06Å), Glu217 (1.70Å), Glu217 (1.72Å), and Glu59 (2.16Å), and carbon hydrogen bond with amino acid Arg297 (2.71Å). It also formed electrostatic π -Cation with amino acids Lys259 (4.03Å) and Arg66 (3.66Å), and hydrophobic π−π T-Shaped bond with amino acids Trp215 (5.10Å), Trp215 (5.38Å), and Trp215 (4.95Å). Luteolin (−30.39 kcal/mol) also had a conventional hydrogen bond with amino acids Lys293 (1.95Å) and Lys293 (2.19Å), carbon hydrogen bond with amino acids Arg298 (2.87Å), and π -donor hydrogen bond with amino acids Arg66 (3.77Å), and Arg66 (2.96Å). It also formed electrostatic attractive charge bond with amino acids Arg66 (3.83Å) and Arg298 (4.83Å), electrostatic π -Cation bond with amino acids Arg66 (3.77Å) and Arg66 (3.00Å), π−π Stacked bond with amino acid Phe294 (5.13Å), π−π T-Shaped bond with amino acids Trp215 (4.92Å) and Trp215 (4.96Å), amide-π stacked bond with amino acids Phe214 (4.95Å) and Trp215 (4.95Å), π-Alkyl bond with an amino acid with Pro212 (4.15Å), Pro212 (4.38Å), Arg297 (4.71Å), Lys69 (4.40Å), and Pro212 (5.36Å). Verapamil (∆G _Binding_: −36.04 kcal/mol) formed a carbon hydrogen bond with amino acids Arg298 (2.82Å), Glu217 (2.48Å), Asp216 (2.53Å), Glu82 (2.98Å) and Glu82 (2.99Å), and π -donor hydrogen bond with amino acid Asn256 (2.93Å). It formed electrostatic π -cation bond with an amino acid with Arg298 (3.44Å) and hydrophobic interactions; π−π T-Shaped bond with amino acid Trp215 (4.88Å), alkyl bond with amino acids Ala258 (4.48Å) and Pro212 (3.94Å), and π -Alkyl bond with amino acid Ala258 (5.05Å). The ranking order of ligands with CAMK2B based on docking score resulted in kaempferol-3-O-glucoside, kaempferol, ellagic acid, quercetin, luteolin, and verapamil.

*Myosin light chain kinase-1:* Quercetin was predicted with the lowest binding energy (∆G _Binding_: −36.92 kcal/mol) and formed conventional hydrogen bond interaction with amino acids Arg456 (2.20Å), His470 (1.86Å), Glu436 (1.61Å), and Trp447 (1.56Å) and hydrophobic π -alkyl bond with amino acids Val463 (4.66Å), Val463 (5.10Å), Ala446 (4.26Å) and Val463 (5.36Å). Luteolin (∆G _Binding_: −28.79 kcal/mol) formed a conventional hydrogen bond with amino acids Trp447 (1.94Å), Arg456 (2.09Å), Val454 (2.07Å), and Glu465 (2.64Å), carbon hydrogen bond with amino acids Trp447 (2.88Å), Arg456 (3.09Å) hydrophobic π -Alkyl bond with amino acids Val463 (4.73Å) and Val463 (4.02Å). Kaempferol (∆G _Binding_: −42.83 kcal/mol) formed a conventional hydrogen bond with amino acids Arg456 (2.18Å), His470 (1.85Å), Trp447 (1.66Å), π -donor hydrogen bond with amino acid Trp447 (2.92Å) and π -alkyl bond with amino acids Val463 (4.56Å), Val463 (5.03Å), Ala446 (4.16Å) and Val463 (5.44Å). Kaempferol-3-o-glucoside (∆G _Binding_: −37.68 kcal/mol) had a conventional hydrogen bond with amino acids Arg456 (2.95Å), Arg456 (1.82Å), Arg456 (2.60Å), Arg456 (2.32Å), Arg456 (2.16Å), Val445 (1.77Å), His470 (1.89Å) and Glu465 (1.79Å), carbon hydrogen bond with amino acids Glu444 (2.50Å), Val445 (2.53Å), Val445 (2.62Å), His470 (2.90Å), Val445 (2.63Å) and π -donor hydrogen bond with amino acid Glu465 (2.76Å). It also formed hydrophobic π -σ bond with amino acid His470 (2.83Å) and π−π T-Shaped bond with amino acid His470 (5.02Å). Ellagic Acid (∆G _Binding_: −32.82 kcal/mol) formed a conventional hydrogen bond with amino acid Trp447 (1.91Å), carbon hydrogen bond with amino acid Ala446 (2.53Å), Trp447 (2.34Å), and Trp447 (2.44Å). It also formed electrostatic π -cation bond with amino acid Arg456 (3.78Å) and Arg456 (4.38Å), electrostatic π -anion bond with amino acid Glu444 (4.67Å), π -alkyl bond with amino acids Val463 (3.97Å), Val463 (5.26Å), Val463 (4.57Å), and Val463 (5.38Å). Verapamil (∆G _Binding_: −33.73 kcal/mol) formed a conventional hydrogen bond with amino acids Arg456 (2.49Å) and Arg456 (2.03Å), carbon hydrogen bond with amino acids Leu449 (2.93Å), Glu450 (2.89Å), Glu465 (2.55Å), Glu465 (2.59Å), Asp481 (2.54Å), and Pro453 (2.63Å) and π -donor hydrogen bond with amino acid Arg480 (4.17Å). It also had an electrostatic π -cation bond with amino acid Arg480 (4.17Å) and alkyl bond with amino acids Leu449 (4.89Å), Val454 (4.56Å), and Ile461 (5.25Å). The ranking order of ligands with MLCK-1 based on docking score is given below: quercetin, luteolin, kaempferol, kaempferol-3-*O*-glucoside, ellagic acid and verapamil.

*Phosphoinositide phospholipase C-gamma-1:* Quercetin again hit the lowest binding energy (∆G _Binding_: −25.63 kcal/mol) for PLCγ1 and formed a conventional hydrogen bond with amino acids Ser612 (2.57Å), Glu548 (2.20Å), Glu548 (1.81Å), and Tyr595 (2.79Å), carbon hydrogen bond with amino acid Ser631 (3.00Å). It also formed hydrophobic interaction; π−π T-Shaped bond with amino acids Phe621 (5.46Å) and Phe621 (5.47Å), π -alkyl bond with amino acids Leu632 (5.19Å), Pro619 (4.48Å) and Pro619 (4.14Å). Luteolin (∆G _Binding_: −29.04 kcal/mol) formed a conventional hydrogen bond with amino acids Ser612 (2.11Å), Glu548 (1.93Å), and Glu548 (1.99Å), π−π T-Shaped bond with amino acids Phe621 (5.57Å), and Phe621 (5.55Å), and π -Alkyl bond with amino acids Leu632 (5.37Å), Leu632 (5.39Å), Pro619 (4.14Å), and Pro619 (4.63Å). Kaempferol (∆G _Binding_: -25.09 kcal/mol) formed a conventional hydrogen bond with amino acid Tyr595 (2.30Å), Glu548 (2.42Å), and Glu548 (1.84Å), π−π T-Shaped bond with amino acid Phe621 (5.48Å), Phe621 (5.33Å), π -Alkyl bond with amino acid Leu632 (5.18Å), Pro619 (4.76Å), and Pro619 (3.99Å). Ellagic acid (∆G _Binding_: −44.22 kcal/mol) had conventional hydrogen bond with amino acids Asp630 (2.14Å), Val628 (2.24Å), Glu667 (2.01Å), Asp634 (1.92Å), and Glu667 (1.75Å), π−π T-Shaped bond with amino acid Phe629 (5.23Å) and Phe629 (4.78Å), π–Alkyl: Leu627 (5.49Å), and Leu627 (4.77Å). Kaempferol-3-O-glucoside formed (∆G _Binding_: −36.39 kcal/mol) formed a conventional hydrogen bond with amino acids Lys666 (1.96Å), Asp630 (1.83Å), Asp634 (1.74Å), Val628 (2.98Å), and Glu667 (1.99Å), carbon hydrogen bond with amino acid Asp634 (2.75Å). It also formed hydrophobic interactions; π−π T-Shaped bond with amino acids Phe629 (5.10Å), and His638 (5.10Å), amide- π Stacked bond with amino acids Thr637 (4.75Å) and His638 (4.75Å), and π -alkyl bond with amino acid Leu627 (4.80Å). Verapamil (∆G _Binding_: −45.13 kcal/mol) formed a conventional hydrogen bond with amino acid Leu632 (2.24Å), carbon hydrogen bond with amino acids Pro619 (2.84Å), Ser631 (2.66Å), Ser631 (2.53Å), Tyr595 (2.34Å), Gln614 (2.76Å), Gly617 (2.93Å), Asp630 (2.71Å), Glu548 (2.59Å), Glu548 (2.66Å), and Glu548 (2.84Å). It formed hydrophobic interaction; π−π T-Shaped bond with amino acid Phe621 (5.22Å), alkyl bond with amino acid Pro619 (4.38Å) and Pro619 (4.85Å) and π–alkyl bond with amino acids Phe551 (4.95Å), Phe621 (5.37Å), Pro619 (4.64Å), and Leu632 (4.70Å). The ranking order of ligands with PLCγ1 based on docking score is given below: quercetin, luteolin, kaempferol, ellagic acid, kaempferol-3-*O*-glucoside, and verapamil.

### 2.4. Isolated Tissue Experimentation

#### 2.4.1. Effects on Isolated Rabbit Jejunum Preparation

**Figure 8:**
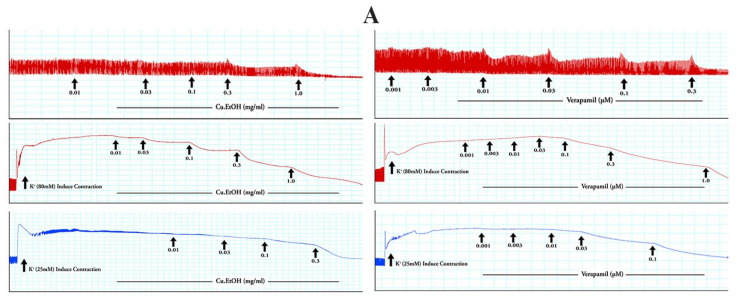

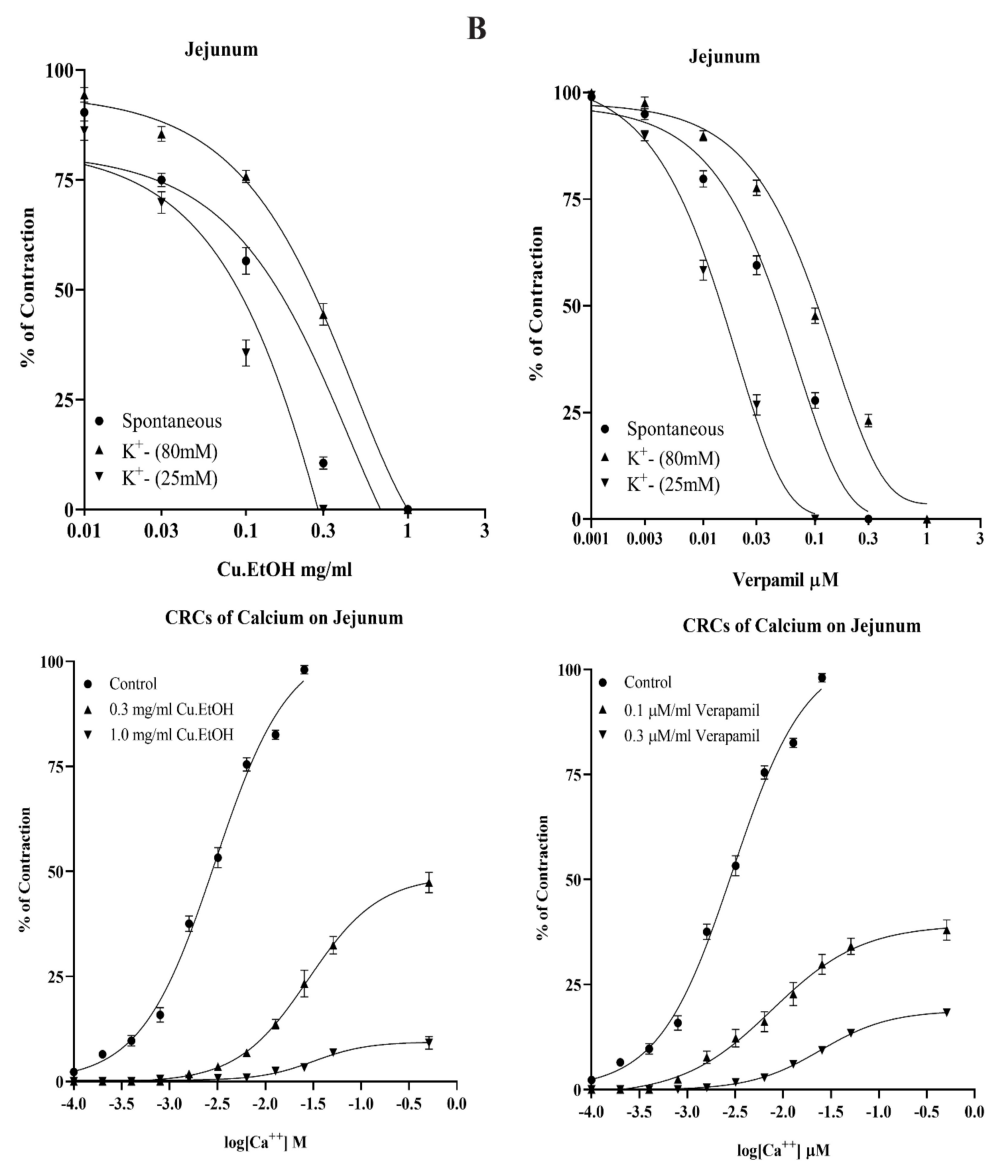
Effect of *C. sativus* seeds extracts (Cu.EtOH) and verapamil on jejunum preparation in respect of spontaneous, K^+^ (80 mM), carbachol, and K^+^ (25 mM) contraction concentration-response curves. (**A**) Physiological responses of Cu.EtOH (**B**) sigmoidal dose-response curve of Cu.EtOH on jejunum preparations (Values are expressed as Mean ± SD, data was analyzed by sigmoidal dose-response curve).

The Cu.EtOH extract was applied to spontaneous contraction of jejunum preparations to examine the antispasmodic effect of the extract. The EtOH extract exerted concentration-dependent spasmolytic effects at 1 mg/mL with complete inhibitory response of K^+^ (80 mM) and K^+^ (25 mM) evoked contractions at dose 3 and 0.3 mg/mL, respectively. When the extract was exposed to rhythmic contraction of jejunum preparations, it showed the concentration-dependent suppression of spontaneous contraction with EC_50_ 0.1362 mg/mL (95% CI: 0.08644 to 0.2136 mg/mL). The EC_50_ of for K^+^ (80 mM) was 0.6268 mg/mL (95% CI: 0.4608 to 0.8921 mg/mL), and 0.1243 mg/mL (95% CI: 0.08309 to 0.1934 mg/mL) for K^+^ (25 mM) was (Figure 8). For confirmation of calcium channel antagonism response of extract, CRCs of calcium were constructed in prior cytosolic calcium-free jejunum preparations in the absence and presence of extract. The extract caused the rightward shifting with suppression of CRCs of calcium at 0.3 and 1 mg/mL, with marked suppression of contractile response calcium CRCs at 1 mg/mL. These results were verified and compared with verapamil, a calcium channel blocker, for possible calcium antagonistic activity of seed extract. Verapamil relaxed spontaneous concentrations, K^+^ (80 mM) and K^+^ (25 mM) induced contractions in jejunum preparations at respective dose 0.3, 1 and 0.1 µM with EC_50_ 0.05876 µM (95% Cl: 0.04740–0.07326 µM), 0.1165 µM (95% CI: 0.1003–0.1355 µM) and 0.01595 µM (95% Cl: 0.01303–0.01956 µM), respectively. Furthermore, CRCs for calcium were built, resulting in completely blocked calcium CRCs at a concentration (0.3 M) comparable to extract (Figure 8), suggesting antagonistic extract activity towards the calcium channels.

#### 2.4.2. Effect on Isolated Rabbit Tracheal Preparations

**Figure 9:**
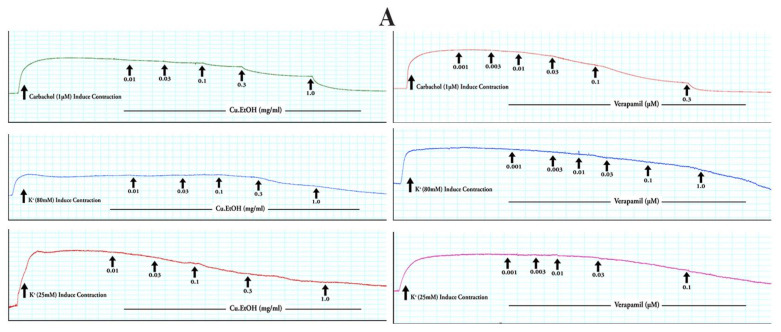

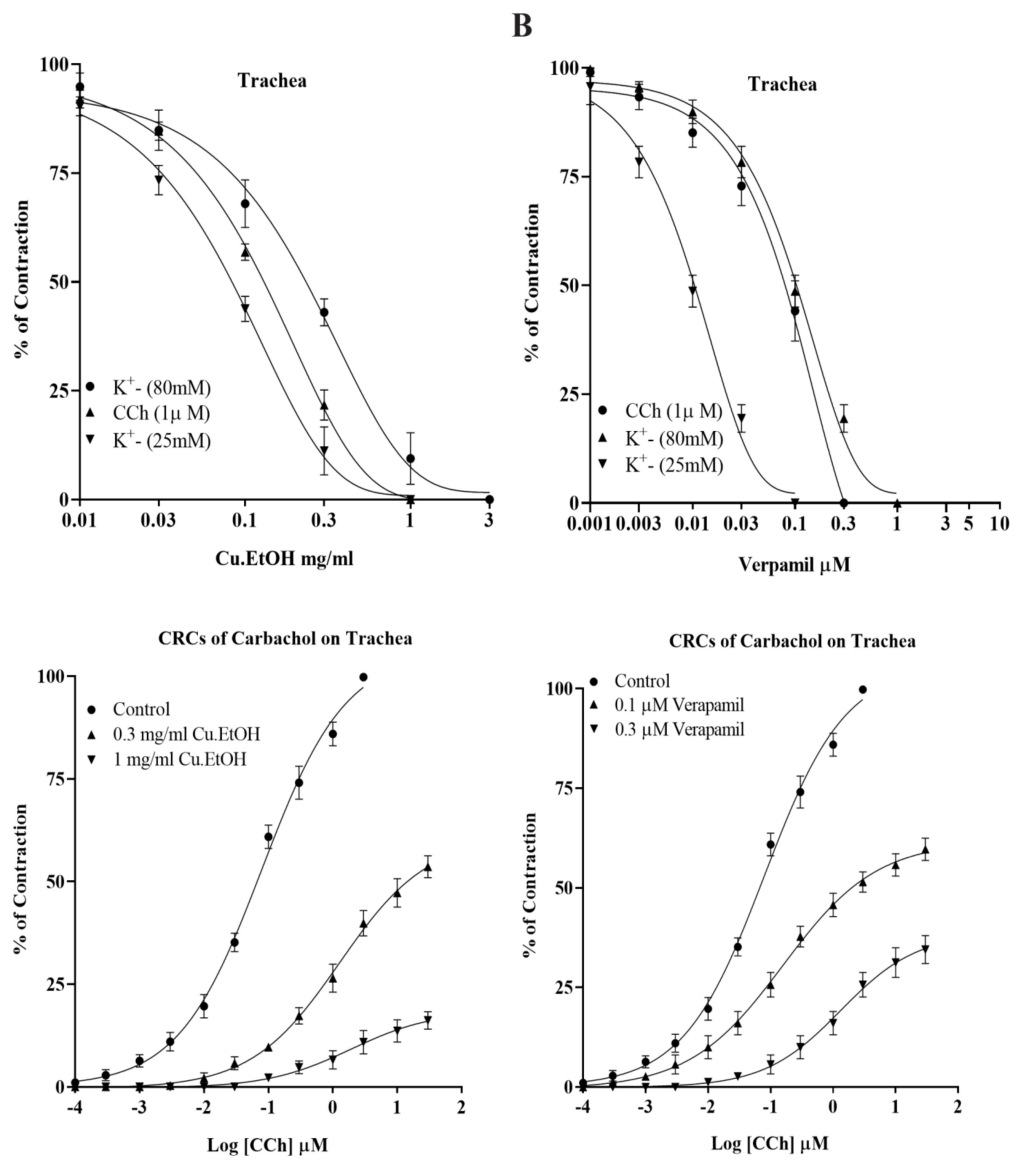
Effect of *C. sativus* seeds extracts (Cu.EtOH) and verapamil on tracheal preparations in respect of spontaneous, K^+^ (80 mM), carbachol, and K^+^ (25 mM) contraction concentration-response curves. (**A**) Physiological responses of Cu.EtOH (**B**) sigmoidal dose-response curve of Cu.EtOH on tracheal bladder preparations (Values are expressed as Mean ± SD, data was analyzed by sigmoidal dose-response curve).

The seeds extract was applied to rabbit tracheal preparations to explore its possible bronchodilator effect. The extract exerted concentration-dependent relaxant effect when exposed to persistent contraction of K^+^ (80 mM) at dose 3 mg/mL with respective EC_50_ 0.2711 mg/mL (95% CI: 0.2058 to 0.3577 mg/mL), while CCh (1 μM) and K^+^ (25 mM) both relaxed at 1 mg/mL with respective EC_50_ 0.1646 mg/mL (95% CI: 0.1342 to 0.2022 mg/mL) and 0.08690 mg/mL (95% CI: 0.06560 to 0.1145 mg/mL) (Figure 9). Carbachol CRCs were constructed in the absence and presence of extract (0.3 and 1 mg/mL) on rabbit tracheal preparation, and the extract caused a noncompetitive rightward shift with suppression of carbachol CRCs. These results were compared with verapamil, which caused the concentration-dependent inhibitory response towards spastic contractions of CCh (1 µM), K^+^ (80 mM) and K^+^ (25 mM) in tracheal preparation at respective dose 0.3, 1 and 0.1 µM with EC_50_ 0.1864 µM (95% CI: 0.1259–0.2945 µM), 0.1212 µM (95% CI: 0.1052–0.1397 µM) and 0.01037 µM (95% CI: 0.008213–0.01307 µM) (Figure 8). The CRCs for CCh were also constructed for verapamil and caused the suppression of CRCs of CCh at dose (1 µM) in a manner like that of extract (Figure 9).

#### 2.4.3. Effect on Isolated Rabbit Urinary Bladder Preparations

**Figure 10:**
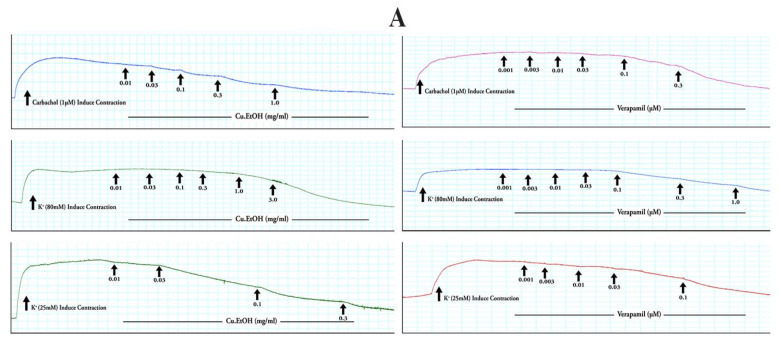

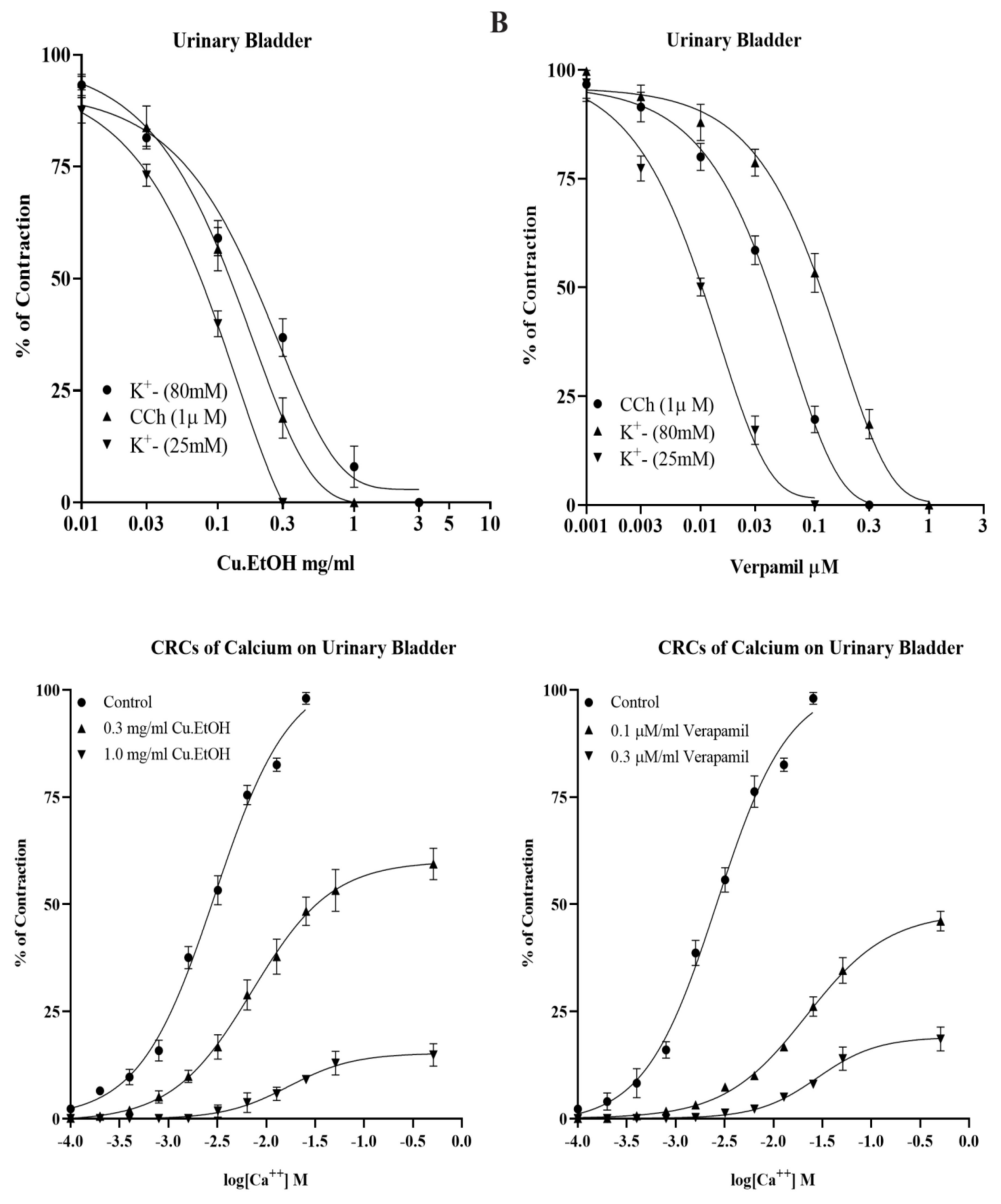
Effect of *C. sativus* seeds extracts (Cu.EtOH) and verapamil on urinary bladder preparations in respect of spontaneous, K^+^ (80 mM), carbachol, and K^+^ (25 mM) contraction concentration-response curves. (**A**) Physiological responses of Cu.EtOH (**B**) sigmoidal dose-response curve of Cu.EtOH on urinary bladder preparations (Values are expressed as Mean ± SD, data was analyzed by sigmoidal dose-response curve).

The extract was applied to rabbit urinary bladder preparations to explore its possible antispasmodic response of smooth muscles. The extract exerted a concentration-dependent inhibitory response when tested on K^+^ (80 mM), CCh (1 μM), and K^+^ (25 mM) induce contractions. The persistent contraction of K^+^ (80 mM), CCh (1 μM) and K^+^ (25 mM) were relaxed at respective doses of 3, 1 and 0.3 mg/mL with EC_50_ 0.1899 mg/mL (95% CI: 0.1483 to 0.2430 mg/mL), 0.1481 mg/mL (95% CI: 0.1107 to 0.1988 mg/mL) and 0.1653 mg/mL (95% CI: 0.1269 to 0.2205 mg/mL) (Figure 10). The calcium channel antagonism response of the extract was confirmed with calcium CRCs, constructed in urinary preparations in the absence and presence of extracts. The extract caused the rightward shifting with suppression of calcium CRCs at 0.3 and 1 mg/mL, with marked suppression of contractile response calcium CRCs at 1 mg/mL. These results were compared with verapamil caused the concentration-dependent inhibitory response of CCh (1 µM), K^+^ (80 mM) and K^+^ (25 mM) evoked contractions at respective dose with 0.3, 1 and 0.1 µM EC_50_ 0.05390 µM (95% CI: 0.04415–0.06608 µM), 0.1425 µM (95% CI: 0.1162–0.1750 µM) and 0.01013 µM (95% CI: 0.007456–0.01370 µM). The calcium CRCs were constructed for verapamil and were caused the suppression of CRCs at a dose (1 µM) similar to *C. sativus* seeds extract (Figure 10).

### 2.5. In Vivo Experiments

#### 2.5.1. Effect on GI Charcoal Meal Intestinal Transit

**Figure 11:**
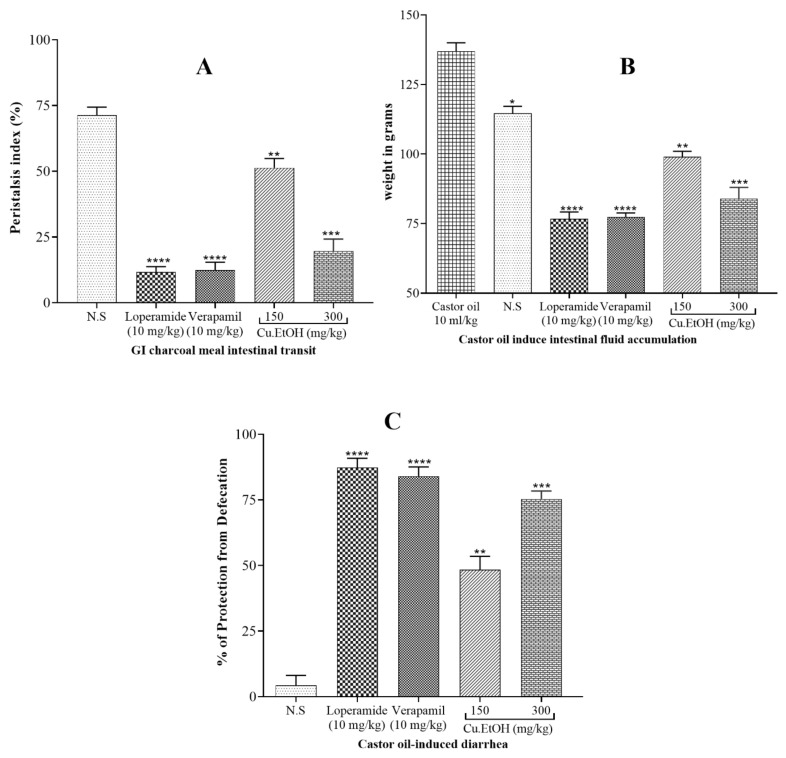
In vivo studies; (**A**) antiperistalsis; (**B**) castor oil induces fluid accumulation inhibition; and (**C**) antidiarrheal effect of seeds extract and verapamil. (Values are expressed as Mean ± SD, data was analyzed by one-way ANOVA followed by Dunnett’s test for in vivo compared to control (normal saline or castor oil group) and *p* < 0.05 was considered significant (* *p* < 0.05, ** *p* > 0.01, *** *p* < 0.001, ****p < 0.0001), N.S: Normal Saline (10 mL/kg).

In propulsive movement assays, the pretreatment of extract of seeds exhibited dose-dependent (150 and 300 mg/kg) decrease (*p* < 0.001 vs. negative control) in charcoal meal propulsive movement. The peristalsis index of extract was 51.4% at dose 150 mg/kg and 19.67% at dose 300 mg/kg like positive controls loperamide (10 mg/kg) and verapamil (10 mg/kg) (Figure 11).

#### 2.5.2. Effect on Castor Oil-Induced Diarrhea

The pretreatment of extract exhibited a dose-dependent (150 and 300 mg/kg) inhibitory effect (*p* < 0.001 vs. negative control) against castor oil-induced diarrhea. The extract showed protection from castor oil-induced diarrhea was 48.3% at 150 mg/kg and 75.4% at 300 mg/kg a significant protection (*p* < 0.001 vs. negative control) from diarrhea, like positive controls loperamide (10 mg/kg) and verapamil (10 mg/kg) (Figure 11).

#### 2.5.3. Effect on Intestinal Fluid Accumulation

In the intestinal fluid accumulation assay, the oral administration of castor oil caused a significant increase (*p*  <  0.001) in fluid accumulation (137  ±  1.7 g) when compared with the normal-saline administered group (112  ±  1.8 g). The pretreatment of seed extract caused a dose-dependent (150 and 300 mg/kg) antisecretory response. The extract administration significantly reduced secretions in the intestine (*p* < 0.001 vs. castor oil group). The weight of fluid for extract was 99.4 ± 1.3 g at 150 mg/kg and 84.3 ± 0.60 g at dose 300 mg/kg, similar to positive controls loperamide (76.5 ± 1.4 g) and verapamil (79.0 ± 0.14 g) (Figure 11).

## 3. Discussion

From ancient times, complementary medicine for treating a wide range of chronic disorders has been recognized as safe and effective. The seeds of *Cucumis sativus* L. have been recognized as traditionally healer seeds and are used to heal multiple ailments of diverse etiology. The LC-MS/MS screening revealed the presence of sterols, anthraquinones, alkaloids, flavones, and flavonoids in *C. sativus* seeds extracts [11]. The major bioactive compounds are stigmasterol, β–sitosterol, scopoletin, 1,4-dicaffeoylquinic acid, luteolin, kaempferol, ferulic acid, epicatechin, ellagic acid, kaempferol-3-*O*-glucoside, quercetin and apigenin. The plant, fruit, or seed containing these compounds may have an antispasmodic effect [26].

In 2007, Hopkins [33] proposed the network pharmacology approach to investigate the cellular mechanism of drugs or ligands. The networks built in network pharmacology reflect the interactive relationship between bioactive compounds, multiple targets, and pathways of bioactive compounds of C. sativus EtOH extract and complex diseases [34]. Recently, network pharmacology has been a novel method to elaborate cellular mechanisms and highlight the potential of herbs’ bioactive compounds [35]. The computational studies of bioactive compounds predicted that they intervene with calcium-mediated signaling and cholinergic synapses to show their antispasmodic activity (Figure 5). The GO and KEGG pathway analysis showed that the bioactive compounds intervened with target genes that are prominent in calcium-mediated signaling and smooth muscle contraction. In molecular docking, kaempferol-3-*O*-glucoside, kaempferol, and quercetin showed the prominent binding affinity for *L*-type voltage-gated calcium ion channels, phosphoinositide phospholipase C, myosin light chain kinase, and calcium/calmodulin kinase similar to verapamil. Hence, it can be assumed that the potent antispasmodic activity of the extract was due to the strong binding affinity of compounds towards target proteins and blockades the signal transduction responsible for contraction.

**Figure 12:**
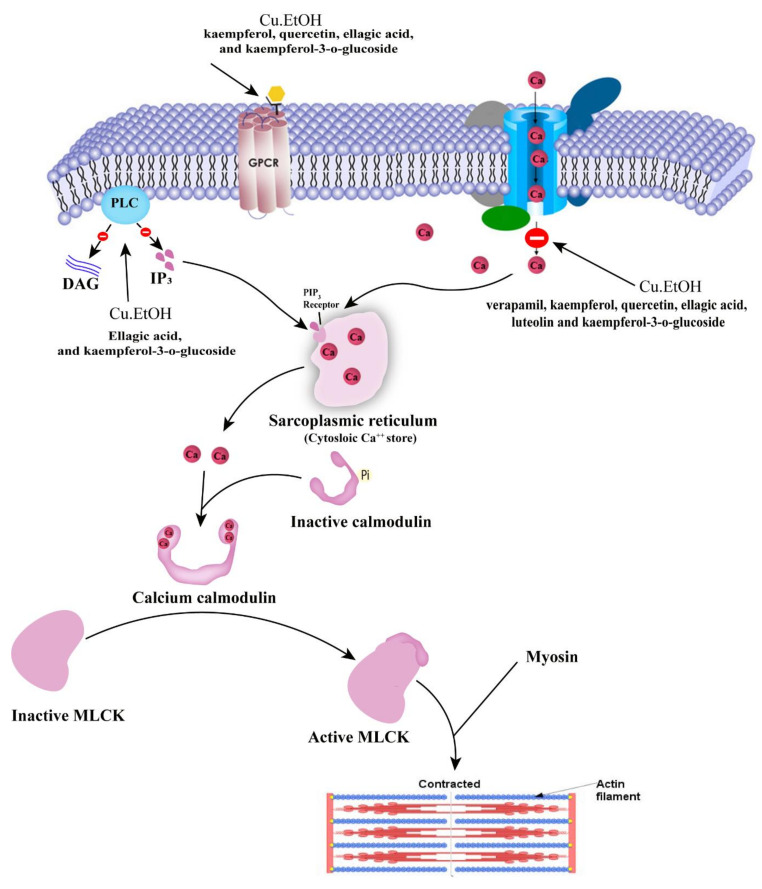
Schematic diagram of proposed mechanism of *C. sativus* seeds extracts on l type voltage gated calcium ion channel, M_3_ muscarinic receptor and phosphoinositide phospholipase C (PLC). (IP3: inositol 1, 4, 5-trisphosphate, DAG: Diacylglycerol MLCK: Myosin Light Chain Kinase).

Numerous physiological mediators (substance P, histamine, prostaglandins, acetylcholine, and 5-HT) regulate the gastrointestinal motor tone [36]. These physiological mediators increase the cytosolic calcium ion levels to facilitate the stimulatory effects, either from an influx of calcium from extracellular fluid or release from cytosolic stores to evoke depolarization of the resting action potential. Any substance blocking this pathway is considered to be an active therapeutic agent in hyperactive gut disorders [37] and is known as an antispasmodic agent [38]. The extract was applied to spontaneous contractions of isolated jejunum for an antispasmodic response, and the spasmolytic effect was found. The extract suppressed the spontaneous contractions of jejunum at dose 1 mg/mL, and this spasmolytic potential of the extract was further investigated against K^+^ (80 mM) and K^+^ (25 mM) spastic contractions [37]. The extract relaxed the K^+^ (80 mM) and K^+^ (25 mM) induced spastic contraction at 1 and 0.3 mg/mL respectively. The K^+^ (80 mM) induced depolarization with an influx of calcium current into the cell, raised in cytosolic calcium ions, and caused the extreme depolarization of membrane action potential to produce a long-lasting persistent contractile response. When repolarization occurs, then the smooth muscle will be relaxed [37]. The EtOH extract relaxed the K^+^ (80 mM) induced contraction at dose 3 mg/mL, obstructing the evoked depolarization, and as a result, membrane action potential repolarizes, thereby blocking the calcium current influx occurred (Figure 5 and Figure 12) [26]. It was expected that the following sequence of events [39] would be inhibited, i.e., (1) cytosolic Calcium ions concentration will decrease; (2) Calcium-calmodulin complex will not form due to absence or less quantity of cytosolic calcium ions to bind with regulatory protein phosphokinase C; (3) as a result activation of myosin light chain kinase (MLCK) will not occur and decrease in myosin light chains (MLCs) phosphorylation; and (4) due to absence of phosphorylation of MLCs, the interaction of actin and MLCs will not occur. As a result, the contractile response in the tissue will not be produced [40], as depicted in computational studies, i.e., kaempferol-3-o-glucoside, ellagic acid, luteolin quercetin, and kaempferol had activities against calcium mediate signaling pathway and intervened with calcium cytosolic concentration. These interaction of bioactive compounds with target genes can cure multiple diseases [41].

The pretreatment of the extract caused suppression of calcium CRCs at doses 0.3 and 1 mg /mL with a rightward shift on jejunum preparation similar to verapamil at doses 0.1 and 0.3 µM [40]. It confirmed the assumptions that the extract acted as CCB and caused the repolarization of the membrane action potential [37]. The agents that relaxed the K^+^ (25 mM) induced contractions are considered as K^+^ channel openers, but CCBs have an an equal tendency to inhibit K^+^ (80 mM) and K^+^ (25 mM) induced contractions [40]. Consequently, seed extract and verapamil completely blocked K^+^ (80 mM) and K^+^ (25 mM) induced contractions [40]. Hence, alterantive medicines had potential therapeutic approaches to treat complex diseases [42]. Previous data suggest that quercetin, epicatechin, rutin, apigenin, and caffeic acid have antispasmodic activity [38,43,44,45]. Amira et al. [46] reported that flavonoids (apigenin, genistein, quercetin, rutin, naringenin, and catechin) had an antispasmodic effect by regulating the gastric tone of the stomach. Thus, the extract’s antispasmodic and antidiarrheal activity may be attributed to the abundant presence of quercetin, epicatechin, rutin, and apigenin.

The antispasmodic activity of the extract was explored on tracheal and urinary bladder tissue preparation with carbachol (1 μM) and K^+^ (80 mM)-induced contractions [47]. The extract exhibited a concentration-dependent relaxation of carbachol (1 μM) evoked contraction at dose 1 mg/mL. The extract showed similar dose-dependent results on tracheal and urinary bladder strips for K^+^ (80 mM)-induced contractions as shown in jejunal tissue preparation. It suppressed the spastic contraction of K^+^ (80 mM) at 3 mg/mL on tracheal and urinary bladder tissue preparations. The EC_50_ value of carbachol (1 μM) for trachea (0.1646 mg/mL) and urinary bladder (0.1481 mg/mL) preparations indicated that extract has muscarinic M_3_ receptor activity as compared to EC_50_ value of K^+^ (80 mM) for trachea (0.2711 mg/mL) and urinary bladder (0.1899 mg/mL) preparations. The anticholinergic response of the extract was confirmed through the rightward shift, with suppression of carbachol CRCs on tracheal preparations at 0.3 and 1 mg/mL, similar to verapamil [41]. Ko et al. [48] studied the broncho-relaxant effect of quercetin, rutin, kaempferol, apigenin, and hesperidin on KCl (30 mM), and carbachol (0.2 µM) induced spastic contraction on isolated guinea pig trachea preparations. Capasso et al. [49] studied the bronchodilator effect of quercetin on rat trachea. Djelili et al. [50] reported that quercetin and rutin had a bronchodilator effect on isolated human bronchus. Chan et al. [51] reported the anti-asthmatic activity of quercetin and rutin. This antimuscarinic response was produced due to blockade of phosphoinositide phospholipase C (PLC), an active member in calcium mediates signaling, as it previously showed in molecular docking, PLC had a strong affinity with kaempferol-3-*O*-glucoside (−5.21 kcal/mol), luteolin (−5.181 kcal/mol), ellagic acid (−4.806 kcal/mol), kaempferol (−3.822 kcal/mol) and quercetin (−3.421 kcal/mol). The activation of the M_3_ muscarinic receptor stimulates the PLC, which hydrolyzes phosphatidylinositol 4,5-bisphosphate into two secondary messengers; inositol 1,4,5-trisphosphate (IP_3_) and diacylglycerol (DAG). IP_3_ stimulates inositol 1,4,5-trisphosphate receptors (IP_3_R) on the sarcoplasmic reticulum to release calcium ions, increasing cytosolic calcium levels. DAG, along with calcium, activates a regulatory protein kinase C (PKC), through which phosphorylation of calmodulin occurs to form a calcium/calmodulin complex. This calcium/calmodulin complex activates another myosin light chain kinase (MLCK) that causes phosphorylation of myosin light chains (MLCs), phosphorylated MLCs, and actin form an interaction network to produce a contractile response (Figure 5 and Figure 12) [37,39]. Hence, the extract exerted its bronchodilator and dysuria action by decreasing cytosolic calcium release from the sarcoplasmic reticulum, thus blocking the signal transduction of the muscarinic receptor pathway of contractile response. When calcium CRCs were constructed, the extract caused suppression with the rightward shift of CRCs on urinary preparation similar to verapamil, as previously acted on jejunum. The carbachol CRCs were constructed on tracheal preparations, which suppressed the carbachol CRCs alike to verapamil.

Diarrhea is characterized as abnormal expulsion of low consistency stool due to disturbance in electrolytes and water transport in the intestine. Castor oil induces changes in electrolyte and water transport in the intestine to cause diarrhea and increase peristaltic movements [52]. Antidiarrheal, antiperistalsis and fluid intestinal accumulation activities of extract were studied. It was found that extract inhibited the effect of castor oil with a significant (*p* < 0.001 vs. negative control) decrease in the wet stool (75.4% at dose 300 mg/kg) as compared to the negative control (normal saline) group, similar to verapamil and loperamide. It also decreased the intestinal transit peristalsis movement of the intestinal tract in GI charcoal meal transit activity. The peristalsis index of the extract was 19.67% at dose 300 mg/kg, similar to loperamide and verapamil. The extract significantly (*p* < 0.001 vs. negative control) reduced the fluid accumulation (84.3 ± 0.60 g at dose 300 mg/kg), similar to loperamide and verapamil. Thus, these results support the in vitro experiments reporting that the extract has an antispasmodic ability because it decreases the intestinal propulsive (peristalsis) and transit movements with blocked rhythmic contractions of the gut. The extract’s antiperistalsis, antisecretory, and antidiarrheal activities were mediated through the involvement of the CCB effect, similar to verapamil. Crowe and Wong [53] reported that loperamide tended to intervene in the calcium-mediated signaling pathway to regulate the intestinal tone. Thus, extract and verapamil decrease intestinal tone in in-vivo activities through a blockade of the calcium channel.

The present study results showed that *C. sativus* seeds of the EtOH extract produced a statistically significant reduction in contractile response of in vitro models, intestinal motility, castor oil-induced diarrhea, and fluid accumulation (electrolytes imbalance) in a dose-dependent manner. The antidiarrheal effect of *C. sativus* seeds of EtOH extract may be induced by inhibiting ricinoleic acid effect on prostaglandin E2 receptors to produce the antiperistalsis and antisecretory antidiarrheal activities [39]. Ricinoleic acid is produced by hydrolyzation of castor oil, which is poorly absorbed from the small intestine that causes an increase in intestinal motility and a decrease in intestinal absorption of water and electrolytes [39]. *C. sativus* EtOH extract abolished intestinal peristalsis movements with a statistically significant delay in feces passage through the bowel. This delay in intestinal transit may increase the opportunity to absorb intestinal fluid from feces, producing dry stool in the bowel [54].

## 4. Materials and Methods

### 4.1. Preparation of Extract

The seeds of *C. sativus* were collected from the pulp of fresh fruits, cultivated in local fields of Multan, Punjab, Pakistan, in May 2018, and rendered free from fruit pulp. Prof Dr. Zafarullah Zafar, Professor, Institute of Pure and Applied Biology, Bahauddin Zakariya University Multan, Pakistan, identified the seeds and fruit (herbarium voucher number: Sp. Pl. 1012/1753, Taxon Id: 200022616). A sample of the specimen was deposited in the herbarium of the same institute with voucher No. Sp. Pl. 1012/1753, Taxon Id: 200022616.

After removing the husk of seeds, the inner portion was ground with an herbal grinder for coarse seeds powder. The ground material was processed in the Soxhlet apparatus for hot maceration with hydroethanolic (70% ethanol and 30% aqueous) solvents and vaporized under reduced pressure at 30 ± 5 °C in a rotary evaporator (Buchi R-200) to obtain a thick brownish yellowish colored extract with an oily profile. The total yield of crude extract was 45%. The extract was shifted to an amber container and stored at −20 °C. The extract was dissolved in distilled water or normal saline and was diluted further in the required concentration on the day of the experiment.

### 4.2. Chemicals

Analytical grade solvents ethanol, methanol, acetonitrile, trifluoroacetic acid, and formic acid were procured from Merck & Co., Darmstadt, Germany. Sodium chloride, potassium dihydrogen phosphate, glucose, calcium chloride, sodium biphosphate, potassium chloride, magnesium sulfate, sodium bicarbonate, magnesium chloride, and ethylene tetra-acetic acid were sodium bicarbonate, and ethylene tetra-acetic were procured from Merck & Co, Germany. Acetylcholine chloride, atropine sulfate, loperamide hydrochloride, verapamil hydrochloride, carbachol chloride were procured from Sigma Chemicals, Co., St. Louis, MO, USA. The analytical grade umbelliferone, stigmasterol, caffeic acid, quinic acid, rutin ferulic acid, epicatechin, scopoletin, 1,4-dicaffeoylquinic acid, quercetin, apigenin-7-*O*-glucuronide, hesperidin, malic acid, p-coumaric acid, luteolin 7-*O*-glucoside and kaempferol were procured from Sigma Chemicals, Co., St. Louis, MO, USA.

### 4.3. Sample Preparation for HPLC and LC-ESI-MS/MS

The extract was dissolved into 1 mL methanol and centrifuged at 14,000 rpm for 10 min. The supernatant was collected and filtered using a syringe filter (0.22 µm).

### 4.4. LC ESI-MS/MS Analysis

The extract was subjected to LC ESI-MS/MS (LTQ XL mass spectrometer, Thermo Electron Corporation, Waltham, MA, USA) [55] to have a tentative screening of potential bioactive constituents. The sample was analyzed with direct injection to the electron spray ionization probe set at a positive and negative ionization mode. The separation of compounds was performed using numerous chromatographic conditions such as mobile phase, flow rate, injection volume, mass range, and column temperature (C_18_, 2.1 × 100 mm, 1.7 μM). The mobile phase is composed of 0.1% formic acid with methanol (Solvent A) and 0.1% formic acid with an acetonitrile (solvent B) at a flow rate of 0.4 mL/min, column temperature 280 °C with a sample injection volume of 8 µL and mass spectrum range 50–1500 *m*/*z* were found optimal for compounds separation. The mobile phase gradient was set as follows: 98% A (*v*/*v*), 98–80% A(*v*/*v*), 80–10% A (*v*/*v*), 10% A (*v*/*v*) and 10–98% A (*v*/*v*) from 0 to 4.0 min, 4.0–7.0 min, 7.0–14.0 min, 14.0–15.0 min, 10–and 15.0–17.0 min accordingly. The optimal ion source parameters for mass spectrometry analysis in positive and negative ionization mode were set as follows: ion spray voltage, 5500 V; curtain gas, 25 psi; nebulizer gas (GS1) and auxiliary gas (GS2), 55 psi; ion source temperature, 400 °C. The negative mode had the same parameters except for ion spray voltage, −5500 V. The isolation of generated ions occurs in an ion trap and is fragmented by collision-induced dissociation (CID) energies ranging 10–45 depending upon the stability of parent precursor ions selected for tandem mass spectrometry. X-Calibur 3.0 (Thermo Scientific, Waltham, MA, USA) was used to acquire and process ESI-MS/MS data.

Identification of Compounds

Major compounds were tentatively identified by comparing their mass spectra previously published data, reference libraries, i.e., Mass Bank of North America (MoNA; http://mona.fiehnlab.ucdavis.edu, accessed on 12 June 2021), and Mass Bank of Europe. The spectra of compounds were compared with high-resolution masses and MS/MS fragmentation of libraries and databases [56]. The structural elucidation was done using ChemDraw 18.0 (PerkinElmer informatics, Waltham, MA, USA)

### 4.5. Quantification of Bioactive Compounds by Using Analytical HPLC-DAD UV/Vis

#### 4.5.1. HPLC Method Optimization

The extract was subjected to reverse-phase HPLC-DAD UV/Vis (Agilent LC) for reconfirmation and quantification of tentatively identified bioactive compounds using LC ESI-MS/MS (stigmasterol, quinic acid, malic acid, epicatechin, caffeic acid, rutin, p-coumaric acid, quercetin, ferulic acid, scopoletin, apigenin, and kaempferol) [57]. For separation of the compounds, various chromatographic conditions such as mobile phase, flow rate, detection wavelength, injection volume, and column temperature according to column size C_18_ column (SB-C18, 4.6 × 150 mm, 5 μM, Agilent Technologies, Waldbronn Germany) were studied and optimized. The mobile phase is composed of solvent A (0.1% trifluoroacetic with methanol) and solvent B (0.1% 0.1% trifluoroacetic with acetonitrile) at flow rate 0.8 mL/min at different detection wavelengths, i.e., 250, 280, and 320 nm with a column temperature 25 °C and a sample injection volume of 8 µL were found to be optimal for compound separation. The linear eluting gradient was set as follows: 95% A (*v*/*v*), 95–90% A(*v*/*v*), 90–70% A (*v*/*v*), 70–10%A (*v*/*v*), 10% A (*v*/*v*) and 10–95% A (*v*/*v*) from 0 to 2.0 min, 2.0–7.0 min, 7.0–12.0 min, 12.0–20.0 min, 20–25 and 25.0–35.0 min accordingly. The data were processed by a comparison with external standards based on retention time and UV spectra.

#### 4.5.2. Validation of the Analytical Method

The method validation was performed under the prescribed guidelines of the International Conference on Harmonization (ICH).

*Linearity*, *limits of detection*, *and quantification:* The dilutions of external standards (stigmasterol, β–sitosterol, scopoletin, 1,4-dicaffeoylquinic acid, luteolin, kaempferol, ferulic acid, epicatechin, ellagic acid, kaempferol-3-*O*-glucoside, quercetin, and apigenin) were prepared for the linearity validation to build the standard curve corresponding to the concentration of each standard and peak area on chromatogram to assessed linearity of the detector response. The following equations determined limits of detection and quantification:

LOD = 3.3σ ÷ S

LOQ = 10σ ÷ S

where σ is the standard deviation of intercept and S is the slope of the linear regression equation.

*Specificity:* A standard solution comprised of external standards was prepared in 100% methanol at a 100 µg/mL concentration. The sample solution was prepared as mentioned in Section 4.3. A volume of 20 μL of standards and sample solutions were injected into the analytical HPLC binary gradient system under chromatographic conditions mentioned in Section 4.5.1. Chromatographic peaks from *C. sativus* sample compared with retention times of external standards for purity of peak and quantified using the calibration curve of each external standard.

*Precision and repeatability:* The precision of the method was determined by injection of external standard solutions on inter-day (triplicate reading) and intra-day (consecutive three days and triplicate readings) under the abovementioned chromatographic conditions and the relative standard deviation (R.S.D) was calculated.

*Accuracy:* The accuracy of the method was assessed through the recovery of external standards in the *C. sativus* seeds EtOH sample. Three different concentrations of external standards (50, 100, and 150 µg) were prepared, then mixed with *C. sativus* sample and made the final volume up to 1 mL, so that the final concentration of external standard and *C. sativus* sample was not disturbed. A volume of 20 μL of prepared solutions was injected into the analytical HPLC binary gradient system under chromatographic conditions mentioned in Section 4.5.1. The sample recovery rate of external standard in *C. sativus* sample was calculated by using the following equation:

% Recovery = (observed concentration ÷ actual concentration) × 100

### 4.6. In Silico Approaches

#### 4.6.1. ADMET and Drug-likeness

The bioactive compounds (stigmasterol, β-sitosterol, scopoletin, 1,4-dicaffeoylquinic acid, luteolin, kaempferol, ferulic acid, epicatechin, ellagic acid, kaempferol-3-o-glucoside, quercetin, apigenin) of *C. sativus* seeds EtOH extract previously identified through LC ESI-MS/MS and quantified with HPLC were subjected for absorption, distribution, metabolism excretion and toxicity (ADMET) in Qikprop module of Maestro (Schrodinger suite 2015), SWISS ADME (http://www.swissadme.ch, accessed on: 10 July 2021) and pkCSM (http://biosig.unimelb.edu.au/pkcsm/prediction, accessed on: 10 July 2021) to evaluate ADMET and drug-likeness parameters [28].

#### 4.6.2. Network Pharmacology Analysis

Network pharmacology was performed according to the method previously reported by Xiao et al. [29]

*Screening potential targets:* The potential protein targets for bioactive compounds were acquired from Drugbank and Swiss Target Prediction datasets. The Gene card, DisGeNET (http://www.disgenet.org/web/DisGeNET, accessed on: 10 July 2021), Pubmed, Online Mendelian Inheritance in Man (OMIM) databases were used to collect targets of gastrointestinal and tracheal disorders with keywords “asthma”; “coughing”; “wheezing”; “diarrhea”; “constipation”; and “irritable bowel syndrome.” The targets of gastrointestinal and tracheal disorders were analyzed in VarElect database (https://ve.genecards.org/, accessed on: 10 July 2021) for a score according to disease phenotype and genetic correlation, and top 150 targets were retained for intersection analysis of bioactive compounds and disease targets via an online toolkit (https://bioinfogp.cnb.csic.es/tools/venny/, accessed on: 10 July 2021) and Venn Diagram were created to show correlative targets of bioactive compounds and disease protein targets. The corelative targets were further preceded for Gene Ontology and KEGG pathway enrichment analysis.

*Construction of network and pathway analyses:* The R studio with packages “BiocManager,” “Clusterprofiler,” and “org.Hs.eg.db” was used for gene ontology and KEGG pathway for analysis and visualization of correlative targets with cutoffs Homo sapiens and *p* < 0.05 for enrichment. The Gene Ontology enrichment network of potential targets, compound target disease (C-T-D) network, Protein–Protein Interaction (PPI) Network, target-pathway (T-P) network map, and Compounds-Targets-Pathways (C-T-P) was constructed in Cytoscape 3.8.0 (U.S. National Institute of General Medical Sciences (NIGMS), USA).

#### 4.6.3. Molecular Docking

Molecular docking studies were performed according to the method previously reported by Sirous et al. [31]

*Ligand Preparation:* The 2D structures of HPLC quantified bioactive compounds were retrieved from PubChem (https://pubchem.ncbi.nlm.nih.gov, accessed on: 14 July 2021) and treated in the LigPrep module of Maestro (Schrodinger suite 2018, Schrödinger, Inc. NY, USA) for ionization, minimization, and optimization of ligands. The Epik tool of this module was used to generate the ionization state of ligands at cellular pH (7.4 ± 0.5) and applied the OPLS3e force field through the module for minimization and optimization of ligands that produce the lowest energy conformer of ligands.

*Protein Preparation*: For molecular docking, the highest resolution X-ray structures of proteins were downloaded from The Protein Databank (RCSB PDB) (https://www.rcsb.org, accessed on: 14 July 2021) and subjected to the Protein preparation wizard of Maestro (Schrodinger suite 2018). This module processed the protein by adding hydrogen atoms to protein structure, removal of solvents (water) molecules, assigning bond orders, creating disulfide bonds and filling missing side chains and loops, and generating protonation state using Epik tool of protein structures for ligands at the cellular level pH (7.4 ± 0.5). After processing protein structures, these structures were optimized using PROPKA under pH 7.0. The OPLS3e force field was utilized to perform restrained minimization for energy minimization and geometry optimization of protein structure.

*Molecular Docking and Receptor grid generation:* The active sites of protein structures for molecular docking were defined in the Receptor Grid Generation module of Maestro (Schrodinger suite 2018). A cubic grid box of each protein was defined with the help of a literature survey and a selection of previously bonded ligands of proteins. The length of the grid box was adjusted to the length of 16 Å. The potential of nonpolar parts of the receptor was decreased to scaling factor 1.0 Å on Vander Waals radius of nonpolar atoms of protein having partial atomic charge cut-off 0.25 Å.

For molecular docking, the prepared ligands and protein structures were subjected to extra precision (XP) mode of Ligand Docking (Glide) module of Maestro (Schrodinger suite 2018) using pre-generated grid file for receptor. 0.80 Å scaling factor was adjusted for Van der Waals radii with a partial charge cut-off of 0.15 Å. The docking results were subjected to the Prime MM-GBSA module to calculate the binding energies of ligands with protein structure using the VSGB solvation model with OPLS3e force field.

*Inhibition Constant (Ki):* The inhibition constant was determined from the binding free energy of ligand previously generated from Prime MM-GBSA, according to the following equation:

∆G = −RT(lnKi) or Ki = e(−∆G/RT)

where ∆G is binding free energy of ligand, R is gas constant (cal.mol^−1^. K^−1^), and T is room temperature (298 Kelvin).

### 4.7. Animals and Housing Conditions

The protocols were permitted from the ethical committee of the Faculty of Pharmacy (vide No. EC/04PhDL/S2018), and experiments were pursued under the guidelines of the Commission of Laboratory Animal Resources [58]. For experimental purposes, albino rabbits (♀/♂, weight: 1.2 to 1.6 kg), Sprague-Dawley rats (♀/♂, weight: 170 to 250 g) and mice (♀/♂, weight: 20 to 30 g) were issued from Faculty of Pharmacy, Bahauddin Zakariya University, Multan. Animals were kept under control conditions (23 ± 2 °C), in faculty animal house followed dark and light cycle with standard food and ad-libitum tap water. Prior to the experiment, the feed was removed from the animals overnight, but free access to the water was maintained. For in vitro experimentation, rabbits were sacrificed with a sharp knife, while mice were deceased with cervical dislocation.

### 4.8. Isolated Tissue Experimentation

Saqib and Janbaz [37] and Gilani et al. [26] previously prescribed methods for isolated tissue experiments in tissue organ bath were used with modification. The physiological response of tissue was recorded through an isotonic transducer (MLT0015) and an isometric transducer (FORT100). Signals were amplified by a data acquisition device Power Lab^®^ (4/25) and visualized in Lab Chart Pro (Version 7). The physiological response was calculated as the percent of contraction, measured immediately before the test drug dose.

#### 4.8.1. Isolated Rabbit Jejunum Preparations

The jejunum tissue was dissected from rabbits, mesenteries were removed from the tissue, and jejunal segments of 2 to 3 cm length were prepared. Each jejunum tissue preparation was suspended in priorly filled Tyrode buffer (pH 7.4) l5 mL tissues organ baths accreted with carbogen (5% CO_2_ and 95% O_2_) at constant temperate 37 °C through a circulating thermoregulator. A preload of 1 g was loaded as tension on jejunum tissue preparation, tissue was permitted to be equilibrated for 25 ± 5 min with drainage of buffer with fresh after 8 ± 2 min, and spontaneous rhythmic contractions were noted prior to the test drug. The spasmolytic and spasmogenic response of extract was studied on equilibrated jejunal preparation [24].

The possible antispasmodic activity of the extract was determined by closing of calcium ion channels through K^+^ (80 mM) and or opening of potassium ion channel through K^+^ (25 mM) [59] evoked contractions on smooth muscles. The extract was applied cumulatively to attain a concentration-dependent inhibitory response of EtOH extract. K^+^ (80 mM) can mediate the contraction in a smooth muscle through cell depolarization by an influx of calcium ions into the cell. The test material that inhibited this K^+^ (80 mM) mediated contraction was considered a calcium channel blocker (CCB).

For calcium antagonism response of the extract, the jejunum preparation was treated with K^+^ (80 mM) three times, then equilibrated for 40 ± 10 min in calcium-free Tyrode buffer solution comprising EDTA (0.12 mM) (replaced with calcium chloride) to deplete intracellular Calcium ions, then again solution was substituted with calcium-free and potassium-rich Tyrode’s solution, included: NaCl (91.0 mM), NaHCO_3_ (11.89 mM), C_6_H_12_O_6_ (5.6 mM), KCl (50.1 mM), Na_2_HPO_4_ (0.43 mM), EDTA (0.12 mM) and MgCl_2_ (2.0 mM). After incubation for 35 ± 10 min, cumulatively, calcium was applied to jejunum preparation to construct control of concentration-response curves (CRCs) of calcium. A gradual rise in contractile response of jejunum preparation indicates that contractions in smooth muscle have a reliability on extracellular calcium ions [37]. When superimposable control CRCs were constructed, jejunum preparation was washed and incubated with extract for 50 ± 10 min; after its incubation, CRCs of calcium were constructed. These CRCs were constructed at different extract concentrations and were compared with control CRCs to determine any possible calcium antagonism.

#### 4.8.2. Isolated Rabbit Tracheal Preparations

The rabbit’s trachea was dissected, fatty and other adherence substances were removed, and about 3 to 4 mm width rings were prepared. The tracheal rings were longitudinally incised to form a tracheal strip; as a result, the central portion of smooth muscles is a sandwich between cartilaginous portions of the strip is exposed. Each isolated tracheal tissue preparation was suspended in priorly filled Kreb’s buffer (pH 7.4), l5 mL tissues organ baths accreted with carbogen (5% CO_2_ and 95% O_2_) at constant temperate 37 °C through a circulating thermoregulator. A preload of 1 g was loaded as tension on tracheal tissue preparation, and tissue was permitted to be equilibrated for 50 ± 10 min with drainage of buffer with fresh after 15 ± 2 min, prior to the test drug.

The possible bronchodilator activity of the extract was determined by K^+^ (80 mM), carbachol (1 µM), and K^+^ (25 mM) evoked contractions on tracheal preparation in a tissue organ bath. The extract was applied cumulatively to attain a concentration-dependent inhibitory response of the extract. The CRCs for carbachol were constructed in the presence and absence of the extract. Cumulatively, carbachol was added to construct control CRCs of carbachol; when the thrice fold contractile response in tissue was constructed, the tissue was washed, and baseline tension was reconstructed. When superimposable control CRCs were constructed, tracheal preparation was incubated with extract for 50 ± 10 min; after its incubation CRCs of carbachol were constructed. These CRCs were constructed at different extract concentrations and compared with control CRCs to determine possible calcium antagonism.

#### 4.8.3. Isolated Urinary Bladder Preparations

The rabbit’s urinary bladder was dissected out. About 2–3 mm wide urinary strips were prepared. Each isolated urinary tissue preparation was suspended in priorly filled Kreb’s buffer (pH 7.4), l5 mL tissues organ baths accreted with carbogen (5% CO_2_ and 95 % O_2_) at constant temperate 37 °C through a circulating thermoregulator. A preload of 1 g was loaded as tension on tracheal tissue preparation, and tissue was permitted to be equilibrated for 50 ± 10 min with drainage of buffer with fresh after 15 ± 2 min, prior to the test drug. The possible antispasmodic activity of the extract was determined by K^+^ (80 mM), carbachol (1 µM), and K^+^ (25 mM) evoked contractions on urinary preparation in a tissue organ bath, and the extract was applied cumulatively to achieve a concentration-dependent inhibitory response.

For calcium antagonism response of extract, the urinary preparation was treated with K^+^ (80 mM) thrice the time, then equilibrated for 40 ± 10 min in calcium-free Kreb buffer solution to depleting intracellular calcium ions. Again, the solution was substituted with calcium-free and potassium-rich Kreb’s solution. After incubation for 35 ± 10 min, cumulatively, calcium was applied to urinary preparation to construct control of concentration-response curves (CRCs) of calcium. A gradual rise in contractile response of urinary preparation indicates that contractions in smooth muscle have reliability on extracellular calcium ions [37]. When superimposable control CRCs were constructed, the urinary preparation was washed and incubated with extract for 50 ± 10 min; after its incubation, CRCs of calcium were constructed. These CRCs were constructed at different extract concentrations and were compared with control CRCs to determine possible calcium antagonism.

### 4.9. In Vivo Experimentation

#### 4.9.1. Evaluation of Maximum Tolerated Dose

Six groups were selected to study the maximum tolerated dose of the extract. Normal saline and five doses of the extract were orally administrated at 50, 100, 150, 200, and 300 mg/kg per day for consecutive 28 days. The body weight, behavioral changes, clinical signs of distress, and mortality of rats were observed for consecutive 28 days [60].

#### 4.9.2. Charcoal Meal GI Transit Test

Saqib and Janbaz [37] previously prescribed the method for GI transit with modifications. Prior to experiments, mice were starved for 12 h and kept in separate cages. Groups were allotted; each group contained five animals. Group I was marked as negative control and normal saline (10 mL/kg) administrated orally. Group II and III were marked as positive controls and received loperamide (10 mg/kg) and verapamil (10 mg/kg) orally. The extract doses were chosen on a trial basis (150 and 300 mg/kg) and orally administrated to Group IV and Group V. After 15 min of treatment, 10 mL/kg of the charcoal meal (contains 20% starch, 10% vegetable charcoal, and 10% gum acacia) in normal saline was given orally. After 30 min, mice were deceased with cervical dislocation and open mice abdomen to remove entire small intestine to measure distance traveled by charcoal meal. The results were calculated as a percentage of the peristaltic index as D_t_/L_i_ × 100, where D_t_ is the distance traveled by charcoal meal, and L_i_ is the length of the small intestine of an animal.

#### 4.9.3. Castor Oil-Induced Diarrhea

Mehmood et al. [61] previously prescribed a method for castor oil-induced diarrhea, which was used with modifications. Prior to experiments, mice were kept in separate cages and starved for 12 h. Groups were allotted; each contained five animals. Group I was marked as a negative control and normal saline (10 mL/kg) was administrated orally. Group II and III were marked as positive controls and received loperamide (10 mg/kg) and verapamil (10 mg/kg) orally. The extract doses were chosen on a trial basis (150 and 300 mg/kg) and orally administrated to Group IV and Group V. After 30 min of treatment, castor oil (10 mL/kg) was orally administered to mice. Cages were inspected for wet diarrhea spots on white paper, and mean defecation per group was calculated 6 h later. The results were expressed as percentage inhibition as (D_c_ − D_t_)/D_c_ × 100, where D_c_ is the mean defecation of the control group, and D_t_ is the mean defecation of the test group.

#### 4.9.4. Intestinal Fluid Accumulation

Mehmood et al. [61] previously prescribed a method for castor oil-induced diarrhea, which was modified. Prior to experiments, mice were kept in separate cages and starved for 12 h. Groups were allotted; each group contained five animals. Group I was marked as a negative control and normal saline (10 mL/kg) was administrated orally. Group II and III were marked as positive controls and received loperamide (10 mg/kg) and verapamil (10 mg/kg) orally. The extract doses were chosen on a trial basis (150 and 300 mg/kg) and orally administered to Group IV to Group V. After 30 min of treatment, castor oil (10 mL/kg) was orally administered to mice, except for mice Group I. After 30 min, mice were deceased with cervical dislocation. Mice abdomen was incised, ligated both ends of small intestine pylorus and caecum and carefully removed from the entire small intestine. The small intestine with intestinal fluid was weighed, and the results were calculated using the formula (Pi/Pm) × 1000, where Pm is the bodyweight of mice and where Pi is the weight of the intestine, and intestinal fluid accumulation values were expressed as weight of fluid (g).

### 4.10. Statistical Analysis

The values of experiments were expressed in mean ± standard deviation (SD), and to determine the median effective concentration (EC_50_) with 95% confidence interval (CI), sigmoidal dose-response graphs were plotted using a nonlinear regression curve fit. The logarithmic sigmoidal dose-response graphs were plotted for concentration-response curves. For in vivo studies, One-way ANOVA was applied, followed by the Dunnett test where *p* < 0.05 was considered significant. Graphpad Prism (Version 8.0) was utilized for all statistical analysis and plotting of graphs

## 5. Conclusions and Future Perspectives

*Cucumis sativus* L. seeds hydroethanolic extract exhibited medicinal effects, besides its nutrition, which may be necessary for improving asthmatic, dysuria, and diarrhea conditions by regulating the contractile response through calcium mediate signaling to the repolarize membrane action potential. In addition, the presence of kaempferol-3-*O*-glucoside, ellagic acid, luteolin, quercetin, and kaempferol may be considered promising bioactive compounds of extract. However, more detailed studies are required to elucidate the possible mechanisms of action and pathways responsible for antispasmodic, antiperistalsis, antidiarrheal, dysuria, and anti-asthmatic capacities of the extract. In conclusion, these results suggest that extract possesses antispasmodic, antiperistalsis, antidiarrheal, dysuria, and anti-asthmatic and provides the preliminary scientific background to the traditional utilization of C. sativus seeds as a remedy for asthma, diarrhea, and dysuria.

## Figures and Tables

**Table 1:** Identification of compounds in the hydroethanolic extract of *Cucumis sativus* L.

Sr. No	Rt (min)	Molecular Weight	Observed MS (*m*/*z*)	Calculated MS (*m*/*z*)	Error (ppm)	Precursor Type	ESI-IT MS/MS (Ions)	Empirical Formula	Proposed Compound	Class
1.	0.83	414.7	413.3785	413.3781	−1	[M−H]−	412.08, 391.33, 365.33, 352.33, 311.25, 297, 285.08, 255.08, 171	C_29_H_50_O	β-Sitosterol	Phytosterols
2.	0.89	312.5	311.3342	311.3335	−2.2	[M−H]−	311, 221, 184, 119	C_20_H_32_O_2_	Arachidic acid	Fatty acids/ω-6 fatty acid
3.	1.62	162.14	161.0252	161.0249	−1.9	[M−H]−	161, 133, 117,	C_9_H_6_O_3_	Umbelliferone	Hydroxycoumarins
4.	2.8	462.36	461.0783	461.0775	−1.7	[M−H]−	461, 285, 175, 151, 133,107	C_21_H_18_O_12_	Luteolin 7-*O* glucuronide	Flavone glucuronide
5.	3.4	458.37	457.0756	457.0749	−1.5	[M−H]−	305, 169, 125	C_22_H_18_O_11_	Epigallocatechin gallate	Catechin gallates
6.	4.06	594.52	593.5036	593.5031	−0.8	[M−H]−	593, 285, 151, 133, 107	C_27_H_30_O_15_	Luteolin 7-*O*-rutinoside	Flavonoid glycosides
7.	4.2	624.54	623.1618	623.1615	−0.5	[M−H]−	623,315, 300, 271, 285, 151	C_28_H_32_O_16_	Narcissin/Isorhmnetin 3-O-rutinoside	Flavonoid glycosides
8.	4.2	462.36	461.073	461.0735	1.1	[M−H]−	461, 285, 257, 229, 175, 113	C_21_H_18_O_12_	Kaempferol-3-*O*-glucuronoside/Kaempferol 3-glucuronide	Flavonoid glycosides
9.	4.28	448.38	447.0943	447.0938	−1.1	[M−H]−	447.09, 284, 285, 271, 284, 253, 179, 151	C_21_H_20_O_11_	Kaempferol-3-*O*-glucoside	Flavonoid glycosides
10.	4.3	178.18	177.05125	177.05134	0.5	[M−H]−	177, 135, 133, 119	C_10_H_10_O_3_	4-Methoxycinnamic acid	Cinnamic acids
11.	4.3	346.37	345.031	345.0312	0.6	[M−H]−	345.52, 300, 226, 221, 206	C_19_H_22_O_6_	Gibberellin A3	Diterpenoids
12.	4.34	478.4	477.1038	477.1034	−0.8	[M−H]−	477,314, 299, 285, 271, 243,151	C_22_H_22_O_12_	Isorhamnetin-3-*O*-glucoside	Flavonoid glycosides
13.	4.63	192.17	191.0357	191.0355	−1	[M−H]−	192, 191, 176, 148, 137, 104	C_10_H_8_O_4_	Scopoletin	Hydroxycoumarins
14.	4.7	432.38	431.0984	431.099	1.4	[M−H]−	431, 285, 284, 255, 227, 183	C_21_H_20_O_10_	Kaempferol-3-*O*-rhamnoside	Flavonoid glycosides
15.	5.2	448.38	447.0933	447.0934	0.2	[M−H]−	445.25, 417.08, 357.17, 327.17, 297, 269, 225, 171	C_21_H_20_O_11_	Orientin	Flavone glucoside
16.	6.2	256.42	255.2325	255.2322	−1.2	[M−H]−	255, 235, 217, 181	C_16_H_32_O_2_	Palmitic acid	Fatty acids
17.	6.3	270.24	269.0425	269.0434	3.3	[M−H]−	241, 225, 197, 157, 133	C_15_H_10_O_5_	Genistein	Isoflavones
18.	6.45	290.27	289.0717	289.0719	0.7	[M−H]−	290, 289, 245, 179, 165.08, 151.25	C_15_H_14_O_6_	Epicatechin	Catechin/Flavonoids
19.	6.6	272.25	271.0632	271.0639	2.6	[M−H]−	271, 177, 151, 119, 107	C_15_H_12_O_5_	Naringenin	Flavanones
20.	7.1	286.24	285.042	285.0415	−1.8	[M−H]−	285, 217, 175, 151, 133,107	C_15_H_10_O_6_	Luteolin	Flavones
21.	7.4	196.16	195.0508	195.0503	−2.6	[M−H]−	75,99, 101,129, 159, 177	C_6_H_12_O_7_	Gluconic acid	Sugar acids and derivatives
22.	7.6	164.16	163.0428	163.0432	2.5	[M−H]−	163.0, 119, 93, 65	C_9_H_8_O_3_	*p*-Coumaric acid	Hydroxycinnamic acids
23.	8.7	610.56	609.1817	609.1826	1.5	[M−H]−	609, 486, 301, 285,242, 151	C_28_H_34_O_15_	Hesperidin	Flavonoid glycosides
24.	8.8	302.19	300.9993	300.9989	−1.3	[M−H]−	300.08, 283, 257.18,201, 207, 172	C_14_H_6_O_8_	Ellagic acid	Tannins
25.	9.9	194.18	193.0509	193.0512	1.6	[M−H]−	193, 178, 149, 134	C_10_H_10_O_4_	Isoferulic acid	Cinnamic acids
26.	9.95	194.18	193.0505	193.0503	−1.0	[M−H]−	181, 164, 133, 118, 114.1, 104	C_10_H_10_O_4_	Ferulic acid	Cinnamic acids
27.	15.51	302.23	301.0359	301.0354	−1.7	[M−H]−	301.08, 286, 273.08, 179, 151, 121,107	C_15_H_10_O_7_	Quercetin	Flavonoids
28.	16.7	286.23	285.0405	285.0406	0.4	[M-H]-	286, 247, 227, 219, 209 151, 142.97, 117	C_15_H_10_O_6_	Kaempferol	Flavanols
29.	16.8	270.24	269.045	269.0455	1.9	[M−H]−	269, 240, 225, 151, 117	C_15_H_10_O_5_	Apigenin	Flavonoids
30.	17.5	516.4	515.013	515.0128	−0.4	[M−H]−	471, 394, 353, 341 327.25, 317,	C_25_H_24_O_12_	1,4-Dicaffeoylquinic acid	Quinic acids
31.	21.4	228.37	227.2018	227.2021	1.3	[M−H]−	227, 209, 67	C_14_H_28_O_2_	Myristic acid	Fatty acids
32.	22.6	430.71	431.3878	431.3883	1.2	[M+H]+	432, 430, 205, 166, 165, 136,	C_29_H_50_O_2_	α-Tocopherol	Fat-soluble vitamins

**Table 2:** Quantification and method validation of bioactive compounds of a hydroethanolic extract of *Cucumis sativus* L. seeds.

Analytes	Λ (nm)	Rt (min)	Linear Regression Data			LOD (µg/mL)	LOQ (µg/mL)	Concentration (µg/g)	Precision (RSD %)		Recovery		Analytes + Extract (µg/g)	
			Range (µg/mL)	Equation	r^2^				Inter Day	Intra Day	Mean	RSD%	50 µg	100 µg
Stigmasterol	250	18.2	7.81−500	y = 3.58x + 5.86	0.9999	0.63	1.93	243.66	1.11	1.67	99.55 ± 1.12	1.13	292.78	341.16
β-sitosterol		19.5	7.81−500	y = 41.589x + 8.04	0.9998	0.56	1.70	317.04	0.44	0.68	99.20 ± 0.54	0.54	365.09	416.97
Scopoletin	280	5.4	7.81−500	y = 50.954x + 6.52	0.9997	0.44	1.33	217.40	0.97	1.60	99.23 ± 1.58	1.59	263.75	315.94
1,4-Dicaffeoylquinic acid		7.2	7.81−500	y = 42.941x + 8.99	0.9994	0.63	1.92	452.18	0.99	1.20	98.5 8± 0.46	0.47	499.45	550.91
Luteolin		13.4	7.81−500	y = 56.949x + 6.10	0.9999	0.31	0.94	617.17	0.99	0.40	98.60 ± 0.88	0.89	665.72	716.35
Kaempferol		19.4	7.81−500	y = 70.954x + 4.82	0.9999	0.20	0.61	783.02	1.67	1.42	100.00 ± 0.82	0.82	831.95	882.09
Ferulic acid		24.9	7.81−500	y = 58.902x + 5.68	0.9999	0.28	0.85	355.35	0.71	1.44	99.58 ± 1.13	1.14	404.45	444.85
Epicatechin	320	4.7	7.81−500	y = 51.969x + 6.77	0.9996	0.39	1.17	370.45	1.33	1.39	99.39 ± 1.51	1.51	418.70	469.41
Ellagic acid		7.4	7.81−500	y = 77.965x + 8.34	0.9998	0.28	0.83	542.71	1.29	0.92	99.70 ± 0.48	0.48	591.11	641.78
Kaempferol-3-*O*-glucoside		11.6	7.81−500	y = 67.572x + 7.34	0.9999	0.32	0.96	457.81	0.48	1.28	99.95 ± 0.06	0.06	505.63	556.55
Quercetin		13.8	7.81−500	y = 61.58x + 4.48	0.9999	0.27	0.81	693.83	1.75	1.13	99.20 ± 0.87	0.88	742.80	792.49
Apigenin		17.7	7.81−500	y = 53.583x + 3.33	0.9999	0.31	0.93	578.93	0.73	0.89	99.53 ± 0.55	0.55	627.26	676.78

**Table 3:** ADMET and drug-likeness profiling of bioactive compounds.

Parameters	1,4-Dicaffeoylquinic Acid	Epicatechin	Ferulic Acid	Beta Sitosterol	Kaempferol-3-O-Glucoside	Ellagic Acid	Luteolin	Apigenin	Stigmasterol	Scopoletin	Quercetin	Kaempferol
Molecular weight (MW)	516.457	290.272	194.187	414.713	448.382	302.197	286.24	270.241	412.698	192.171	302.24	286.24
QPlogP o/w	0.613	0.469	1.371	7.622	−0.748	−1.295	0.926	1.605	7.737	0.854	0.367	1.041
QPlog S	−4.157	−2.616	−1.864	−8.638	−2.452	−1.918	−3.067	−3.332	−9.204	−1.742	−2.909	−3.157
QPlog HERG	−4.992	−4.732	−2.239	−4.684	−4.932	−3.842	−5.023	−5.114	−4.938	−3.781	−5.109	−5.201
QPP Caco	0.259	53.201	63.536	3381.654	9.636	7.907	40.856	114.487	3379.988	632.797	18.199	51.24
QPlog BB	−5.175	−1.882	−1.175	−0.354	−2.937	−2.395	−1.955	−1.446	−0.313	−0.571	−2.419	−1.893
QPP MDCK	0.084	20.76	31.988	1846.188	3.275	2.645	15.606	47.531	1845.206	301.676	6.511	19.934
QPlog Kp	−6.788	−4.728	−3.674	−1.651	−5.636	−6.701	−4.888	−3.989	−1.747	−3.065	−5.544	−4.641
VDss (human)	1.96	1.027	−1.367	0.193	1.444	0.375	1.153	0.822	0.178	0.034	1.559	1.274
CNS permeability	−3.804	−3.298	−2.612	−1.705	−3.908	−3.533	−2.251	−2.061	−1.652	−2.32	−3.065	−2.228
Bioavailability Score	-	0.55	0.85	-	0.17	0.55	0.55	0.55	-	0.55	0.55	0.55
QPlog Khsa	−0.738	−0.412	−0.612	2.077	−0.751	−0.658	−0.198	−0.039	2.169	−0.481	−0.343	−0.191
Percent Human Oral Absorption	0	60.584	67.241	100	14.261	35.438	61.205	73.192	100	82.085	51.649	63.637
GI absorption	Low	High	High	High	Low	High	High	High	High	High	High	High
*P*-glycoprotein substrate	Yes	Yes	No	No	No	No	No	No	No	No	No	No
*P*-glycoprotein I inhibitor	No	No	No	Yes	No	No	No	No	Yes	No	No	No
*P*-glycoprotein II inhibitor	No	No	No	Yes	No	No	No	No	Yes	No	No	No
Primary metabolites	6	7	2	3	7	4	4	3	5	2	5	4
CYP1A2 inhibitor	No	No	No	No	No	Yes	Yes	Yes	No	Yes	Yes	Yes
CYP2C19 inhibitor	No	No	No	No	No	No	No	No	No	No	No	No
CYP2C9 inhibitor	No	No	No	No	No	No	No	No	No	No	No	No
CYP2D6 inhibitor	No	No	No	No	No	No	Yes	Yes	No	No	Yes	Yes
CYP3A4 inhibitor	Yes	No	No	Yes	No	No	Yes	Yes	Yes	No	Yes	Yes
Total Clearance (mL/min/kg)	−0.062	0.183	0.623	0.628	0.462	0.537	0.495	0.566	0.618	0.73	0.407	0.477
Renal OCT2 substrate	No	No	No	No	No	No	No	No	No	No	No	No
AMES toxicity	No	No	No	No	No	No	No	No	No	No	No	No
Oral Rat Acute Toxicity (LD50)	2.543	2.428	2.282	2.552	2.546	2.399	2.455	2.45	2.54	1.95	2.471	2.449
Oral Rat Chronic Toxicity (LOAEL) (mg/kg/day)	3.889	2.5	2.065	0.855	4.53	2.698	2.409	2.298	0.872	1.378	2.612	2.505
Hepatotoxicity	No	No	No	No	No	No	No	No	No	No	No	No
Skin Sensitization	No	No	No	No	No	No	No	No	No	No	No	No
Lipinski violations	-	0	0	-	2	0	0	0	-	0	0	0
Ghose violations	-	0	0	-	0	0	0	0	-	0	0	0
Veber violations	-	0	0	-	1	1	0	0	-	0	0	0
Egan violations	-	0	0	-	1	1	0	0	-	0	0	0
Muegge violations	-	0	1	-	3	0	0	0	-	1	0	0

MW: Molecular weight of the molecule in Dalton, 130.0–500.0; QPlogPo/w: Predicted octanol/water lipophilicity partition coefficient, −2 to 6.5; QPlogS: Predicted aqueous solubility: −6.5 to 0.5; QPlogHERG: Predicted IC_50_ for blockage of HERG K^+^ channels greater than −5; QPPCaco: Predicted apparent Caco-2 cell, model for gut-blood barrier, permeability in nm/s, poor if <25 and great if >500; QPlogBB: Predicted brain/blood partition coefficient, −3 to 1.2; QPPMDCK: Predicted apparent MDCK cell permeability in nm/sec predicting non-active transport across blood brain barrier, <25 poor and >500 great; Primary metabolites: Number of Primary metabolites,1–8; QPlogKp: Predicted skin permeability, −8.0 to −1.0 cm/s; QPlogKhsa: Predicted human serum albumin binding, −1.5 to 1.5; VDDs: volume of distribution, low < −0.15, high > 0.45; CNS permeability: greater than -2 able to penetrate.

**Table 4:** Binding energies (kcal/mol) of compounds with voltage-gated calcium channel beta2a, calcium/calmodulin-dependent protein kinase IIB, Myosin light chain kinase-1, and phosphoinositide phospholipase C-Gamma-1 calculated by Prime MMGBSA.

Name	Docking Score	∆ G_Binding_	Log K_i_ (µMolar)	∆ G_Coulomb_	∆ G_Covalent_	∆ G_Hbond_	∆ G_Lipophilic_	∆G_Solv GB_	∆ G_vdW_	Residue-Ligand Interactions with Distance (Å)	
										Hydrogen Bonds	Electrostatic/Hydrophobic Bonds
**Voltage-gated calcium channel beta2a (VGCC, PDB:1T0J)**											
Kaempferol-3-***O***-glucoside	−5.21	−36.36	−12.56	−19.31	0.93	−3.68	−8.18	28.36	−33.52	**Conventional Hydrogen Bond**: Lys254 (1.80), Arg424 (3.04), Asp251 (1.90), Asp319 (1.74), Ile261 (2.88) **Carbon Hydrogen Bond**: Lys254 (3.03), Asp251 (2.91), Asp319 (3.06)	**πAlkyl**: Ile263 (4.40), Ile263 (4.75), Arg265 (5.41)
Luteolin	−5.181	−29.94	−9.77	−23.33	1.24	−1.53	−3.41	22.59	−24.63	**Conventional Hydrogen Bond**: Arg227 (2.41), Val109 (1.78), Glu381 (1.73)	**Electrostatic π Anion** Asp384 (4.00), Asp384 (3.53) **π−π T-shaped**: Tyr402 (5.79)
Ellagic acid	−4.806	−35.03	−11.98	−22.11	1.12	−1.51	−11.31	26.81	−27.61	**Conventional Hydrogen Bond**: Phe92 (1.95), Arg227 (1.98), Tyr40 (2.89) **Carbon Hydrogen Bond**: Asp91 (2.87), Arg227 (2.99)	**π−Lone pair**: Val109 (2.80) **π−π Stacked**: Tyr402 (5.40) πAlkyl: Lys110 (4.87), Val109 (5.29), Lys110 (5.49), Ala405 (5.33)
Kaempferol	−3.822	−34.67	−11.83	−20.81	0.31	−1.36	−6.91	20.73	−23.84	**Conventional Hydrogen Bond**: Asp91 (2.73), Glu381 (1.81), Val109 (1.75)	**π−π T-shaped**: Phe92 (5.30)
Quercetin	−3.421	−26.09	−8.10	−15.86	0.46	−3.5	−4.44	16.35	−18.58	**Conventional Hydrogen Bond**: Lys247 (2.09), Lys254 (1.93), Arg265 (1.96), Arg424 (2.14), Asp319 (1.71)	**π−Alkyl**: Lys247 (5.30), Ile263 (5.49)
Verapamil	−2.351	−42.52	−15.24	−16.52	1.27	−1.14	−14.39	33.32	−44.34	**Conventional Hydrogen Bond**: Arg227 (2.65) **Carbon Hydrogen Bond**: Tyr402 (2.72), Asp384 (2.54), Tyr402 (2.69), Glu111 (2.61), Ser330 (2.75), Pro336 (2.49), Glu381 (2.67), Ser382 (2.43)	**Electrostatic Attractive Charge**:Asp384 (4.51) **π−Cation**: Arg227 (4.00) **π−Anion**: Asp384 (3.66) **Alkyl**: Ala335 (4.32), Ala405 (3.99), Lys110 (4.24), Pro326 (5.46), Ile338 (4.46) **π−Alkyl**: Phe92 (5.37), Lys110 (5.11), Ala409 (4.44))
**Calcium/calmodulin-dependent protein kinase IIB (CAMK2B, PDB: 3BHH)**											
Kaempferol-3-***O***-Glucoside	−6.39	−41.23	−14.68	−39.64	3.62	−6.08	−6.07	43.77	−32.21	**Conventional Hydrogen Bond**: Arg187 (1.91), Asn256 (2.07), Lys138 (2.17), Lys227 (1.95), Lys227 (1.99), Glu189 (1.78), Glu140 (1.86) **Carbon Hydrogen Bond**: Glu189 (2.82), Glu189 (2.62)	**Electrostatic π−Cation**: Arg298 (4.27), Arg298 (3.18)
Kaempferol	−5.72	−36.84	−12.77	−22.11	4.46	−1.9	−10.67	25.72	−26.29	**Conventional Hydrogen Bond**: Trp215 (1.86), Glu217 (1.74), Phe294 (2.13) **πDonor Hydrogen Bond**: Trp215 (2.44)	**Electrostatic π−Cation**: Arg66 (3.87), Arg298 (4.37) **π−π T-Shaped**: Trp215 (4.32) **π−Alkyl**: Arg297 (4.94), Arg298 (4.83)
Ellagic Acid	−5.231	−18.31	−4.72	−26.11	2.2	−4.38	−2.8	36.58	−23.57	**Conventional Hydrogen Bond**: Asn256 (2.69), Arg66 (2.59), Arg297 (2.09), Glu59 (2.02), Leu300 (2.02), Glu59 (1.89), Leu300 (2.79) **Carbon Hydrogen Bond**: Arg297 (2.66)	**Electrostatic π−Cation**: Lys259 (4.40), Lys259 (3.50 **Electrostatic π−Anion**: Glu83 (3.77), Glu83 (3.94), Glu83 (3.29
Quercetin	−4.999	−30.78	−10.14	−29.96	3.95	−3.98	−4.18	36.29	−27.17	**Conventional Hydrogen Bond**: Arg66 (2.06), Glu217 (1.70), Glu217 (1.72), Glu59 (2.16) **Carbon Hydrogen Bond**: Arg297 (2.71)	**Electrostatic π−Cation**: Lys259 (4.03), Arg66 (3.66) **π−π T-Shaped**: Trp215 (5.10), Trp215 (5.38), Trp215 (4.95)
Luteolin	−4.885	−30.39	−9.97	−24.26	1.96	−3.59	−4.59	28.74	−28.33	**Conventional Hydrogen Bond**: Lys293 (1.95), Lys293 (2.19), **Carbon Hydrogen Bond**: Arg298 (2.87) π −Donor Hydrogen Bond: Arg66 (3.77), Arg66 (2.96)	**Electrostatic Attractive Charge**: Arg66 (3.83), **Electrostatic π−Cation**: Arg66 (3.77), Arg66 (3.00) **π−π Stacked**: Phe294 (5.13) **π−π T-Shaped**: Trp215 (4.92), Trp215 (4.96) **Amide−π Stacked**: Phe214 (4.95), Trp215 (4.95) **π−Alkyl**: Pro212 (4.15), Pro212 (4.38), Arg297 (4.71), Lys69 (4.40), Pro212 (5.36)
Verapamil	−1.537	−36.04	−12.42	26.1	−0.27	−1.06	−15.28	1.48	−44.54	**Carbon Hydrogen Bond**: Arg298 (2.82), Glu217 (2.48), Asp216 (2.53), Glu82 (2.98), Glu82 (2.99) **π −Donor Hydrogen Bond**: Asn256 (2.93)	**Electrostatic π−Cation**: Arg298 (3.44) **π−π T-Shaped**: Trp215 (4.88) **Alkyl**: Ala258 (4.48), Pro212 (3.94) π−Alkyl: Ala258 (5.05)
**Myosin light chain kinase-1 (MLCK-1, PDB: 6C6M)**											
Quercetin	−6.336	−36.92	−12.81	−35.65	4.07	−2.65	−9.55	31.81	−23.81	**Conventional Hydrogen Bond**: Arg456 (2.20), His470 (1.86), Glu436 (1.61), Trp447 (1.56)	**π−Alkyl**: Val463 (4.66), Val463 (5.10), Ala446 (4.26), Val463 (5.36)
Luteolin	−5.319	−28.79	−9.27	−23.92	4.03	−2.44	−3.17	26.21	−28.28	**Conventional Hydrogen Bond**: Trp447 (1.94), Arg456 (2.09), Val454 (2.07), Glu465 (2.64) **Carbon Hydrogen Bond**: Trp447 (2.88), Arg456 (3.09)	**π−Alkyl**: Val463 (4.73), Val463 (4.02)
Kaempferol	−5.196	−42.83	−15.37	−34.11	2.02	−2.16	−9.6	30.55	−28.39	**Conventional Hydrogen Bond**: Arg456 (2.18), His470 (1.85), Trp447 (1.66) **π −Donor Hydrogen Bond**: Trp447 (2.92)	**π−Alkyl**: Val463 (4.56), Val463 (5.03), Ala446 (4.16), Val463 (5.44)
Kaempferol-3-***O***-Glucoside	−5.184	−37.68	−13.14	−52.04	8.12	−4.28	−8.2	51.95	−32.55	**Conventional Hydrogen Bond**: Arg456 (2.95), Arg456 (1.82), Arg456 (2.60), Arg456 (2.32), Arg456 (2.16), Val445 (1.77), His470 (1.89), Glu465 (1.79) **Carbon Hydrogen Bond**: Glu444 (2.50), Val445 (2.53), Val445 (2.62), His470 (2.90), Val445 (2.63) **π −Donor Hydrogen Bond**: Glu465 (2.76)	**π−σ**: His470 (2.83) **π−π T-Shaped**: His470 (5.02)
Ellagic Acid	−4.453	−32.82	−11.02	−9.37	1.84	−1.53	−9.78	18.6	−30.41	**Conventional Hydrogen Bond**: Trp447 (1.91) **Carbon Hydrogen Bond**: Ala446 (2.53), Trp447 (2.34), Trp447 (2.44)	**Electrostatic π−Cation**: Arg456 (3.78), Arg456 (4.38) **Electrostatic π−Anion**: Glu444 (4.67) **π−Alkyl**: Val463 (3.97), Val463 (5.26), Val463 (4.57), Val463 (5.38)
Verapamil	−2.844	−33.73	−11.42	7.71	7.06	−1.13	−14.36	4.17	−36.53	**Conventional Hydrogen Bond**: Arg456 (2.49), Arg456 (2.03) **Carbon Hydrogen Bond**: Leu449 (2.93), Glu450 (2.89), Glu465 (2.55), Glu465 (2.59), Asp481 (2.54), Pro453 (2.63) **π −Donor Hydrogen Bond**: Arg480 (4.17)	**Electrostatic π−Cation**: Arg480 (4.17) **Alkyl**: Leu449 (4.89), Val454 (4.56), Ile461 (5.25)
**Phosphoinositide Phospholipase C-Gamma-1 (PLCγ1, PDB: 4EY0)**											
Quercetin	−9.119	−25.63	−7.90	−15.74	2.03	−2.81	−10.72	35.42	−32.32	**Conventional Hydrogen Bond**: Ser612 (2.57), Glu548 (2.20), Glu548 (1.81), Tyr595 (2.79) **Carbon Hydrogen Bond**: Ser631 (3.00)	**π−π T-Shaped**: Phe621 (5.46), Phe621 (5.47) **π−Alkyl**: Leu632 (5.19), Pro619 (4.48), Pro619 (4.14)
Luteolin	−8.941	−29.04	−9.38	−21.27	2.61	−3.06	−9.3	36.96	−32.96	**Conventional Hydrogen Bond**: Ser612 (2.11), Glu548 (1.93), Glu548 (1.99)	**π−π T-Shaped**: Phe621 (5.57), Phe621 (5.55) **π−Alkyl**: Leu632 (5.37), Leu632 (5.39), Pro619 (4.14), Pro619 (4.63)
Kaempferol	−8.159	−25.09	−7.67	−16.35	1.09	−2.55	−10.81	36.25	−30.94	**Conventional Hydrogen Bond**: Tyr595 (2.30), Glu548 (2.42), Glu548 (1.84)	**π−π T-Shaped**: Phe621 (5.48), Phe621 (5.33) **π−Alkyl**: Leu632 (5.18), Pro619 (4.76), Pro619 (3.99)
Ellagic Acid	−5.668	−44.22	−15.98	−26.45	2.36	−4.11	−11.4	25.55	−26.49	**Conventional Hydrogen Bond**: Asp630 (2.14), Val628 (2.24), Glu667 (2.01), Asp634 (1.92), Glu667 (1.75)	**π−π T-Shaped**: Phe629 (5.23), Phe629 (4.78) **π−Alkyl**: Leu627 (5.49), Leu627 (4.77)
Kaempferol-3-***O***-Glucoside	−5.566	−36.39	−12.57	−31.94	2.91	−4.4	−8.27	45.71	−37.77	**Conventional Hydrogen Bond**: Lys666 (1.96), Asp630 (1.83), Asp634 (1.74), Val628 (2.98), Glu667 (1.99) **Carbon Hydrogen Bond**: Asp634 (2.75)	**π−π T-Shaped**: Phe629 (5.10), His638 (5.10) **Amide−π Stacked**: Thr637 (4.75), His638 (4.75) **π−Alkyl**: Leu627 (4.80)
Verapamil	−4.069	−45.13	−16.37	−46.51	10.09	−0.67	−24.7	58.56	−39.94	**Conventional Hydrogen Bond**: Leu632 (2.24) **Carbon Hydrogen Bond**: Pro619 (2.84), Ser631 (2.66), Ser631 (2.53), Tyr595 (2.34), Gln614 (2.76), Gly617 (2.93), Asp630 (2.71), Glu548 (2.59), Glu548 (2.66), Glu548 (2.84)	**π−π T-Shaped**: Phe621 (5.22) **Alkyl**: Pro619 (4.38), Pro619 (4.85) **π−Alkyl**: Phe551 (4.95), Phe621 (5.37), Pro619 (4.64), Leu632 (4.70)

∆L_Binding:_ Binding free energy, Log Ki: Logarithmic of Inhibition Constant (K_i_), ∆o_Coulomb_: Coulomb binding energy, ∆o_Covalent_: Covalent binding energy ∆o_H_: Hydrogen ing energy, ∆n_Lipophilic_: Lipophilic binding energy, ∆L_Solv GB_: Generalized born electrostatic solvation energy ∆G_vdW_: Van der Waals forces energy, all these energies contribute to Binding free energy (∆c_Binding_).

## Data Availability

Data is contained within the article.

## Supplementary Materials

The following are available online at https://www.mdpi.com/article/10.3390/ph14111197/s1, Figure S1: Venn Diagram between target genes and compounds, Figure S2: Protein-protein interaction of target genes of respiratory and gastrointestinal disorders, Table S1: Precision validation of analytical method C. sativus EtOH extract, Table S2: Accuracy validation of analytical method of C. sativus EtOH extract through % recovery method, Table S3: Gastrointestinal and respiratory target genes of EtOH extract of *Cucumis sativus* L. seeds, Table S4: Top 15 GO Biological process of bioactive compounds of hydroethanolic extract of *C. sativus* for gastrointestinal and respiratory target genes, Table S5: Top 15 KEGG pathway of bioactive compounds of hydroethanolic extract of *C. sativus* for gastrointestinal and respiratory target genes, Table S6: Network analysis of target genes interaction with phytoconstituents, Table S7: Network analysis of target genes interaction with phytoconstituents and GO biological process (BP), Table S8: Network analysis of target genes interaction with phytoconstituents and KEGG pathway.

## Abbreviations

BP	Biological process
C-T-D	Compound and targeted disease genes network
C-T-P	Compound, targeted disease genes and pathway network
CAMK2B	Calcium/calmodulin-dependent protein kinase IIB
CCB	Calcium Channel blocker
CCh	Carbachol
CRC	Concentration-response curves
GO	Gene Ontology
KEGG	Kyoto Encyclopedia of Genes and Genomes
Log Ki	Logarithmic of Inhibition Constant (K_i_)
MM-GBSA	molecular mechanics energies combined generalized Born and surface area
MLCK-1	Myosin light chain kinase-1
Cu.EtOH	*C. sativus* seeds hydroethanolic extract
VGCC	Voltage-gated calcium channel β2a
