# Supplementary material for: Cucumis sativus L. Seeds Ameliorate Muscular Spasm-Induced Gastrointestinal and Respiratory Disorders by Simultaneously Inhibiting Calcium Mediated Signaling Pathway
Source: Pharmaceuticals (Basel). 2021 Nov 22;14(11):1197. doi: 10.3390/ph14111197 (PMC8624829; doi:10.3390/ph14111197)
Supplement: Supplementary file 1 [file pharmaceuticals-14-01197-s001.zip › pharmaceuticals-1414234-supplementary.pdf]

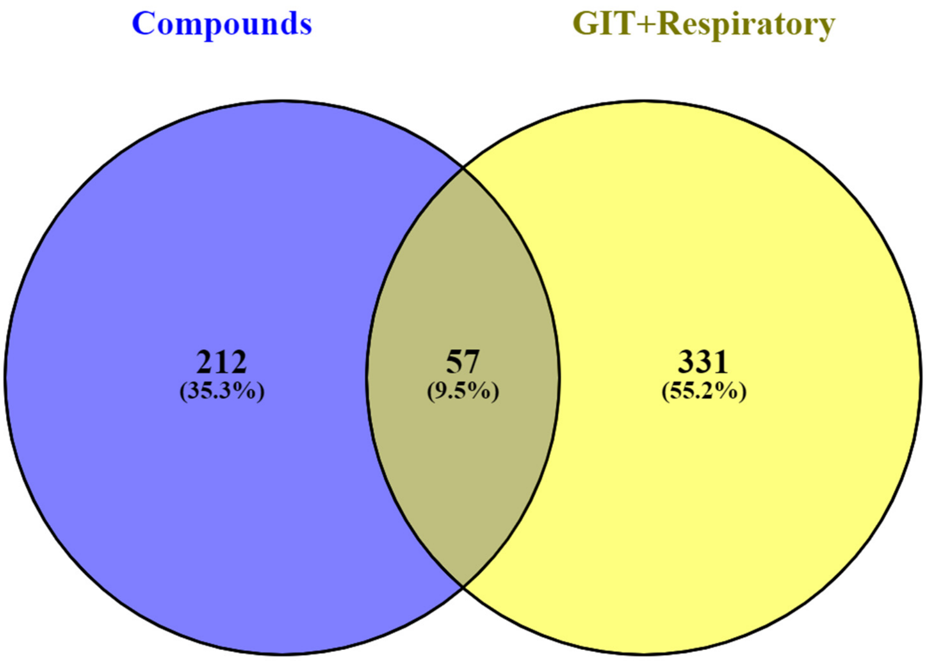

Figure S1. Venn Diagram between pathogenic genes and compounds.

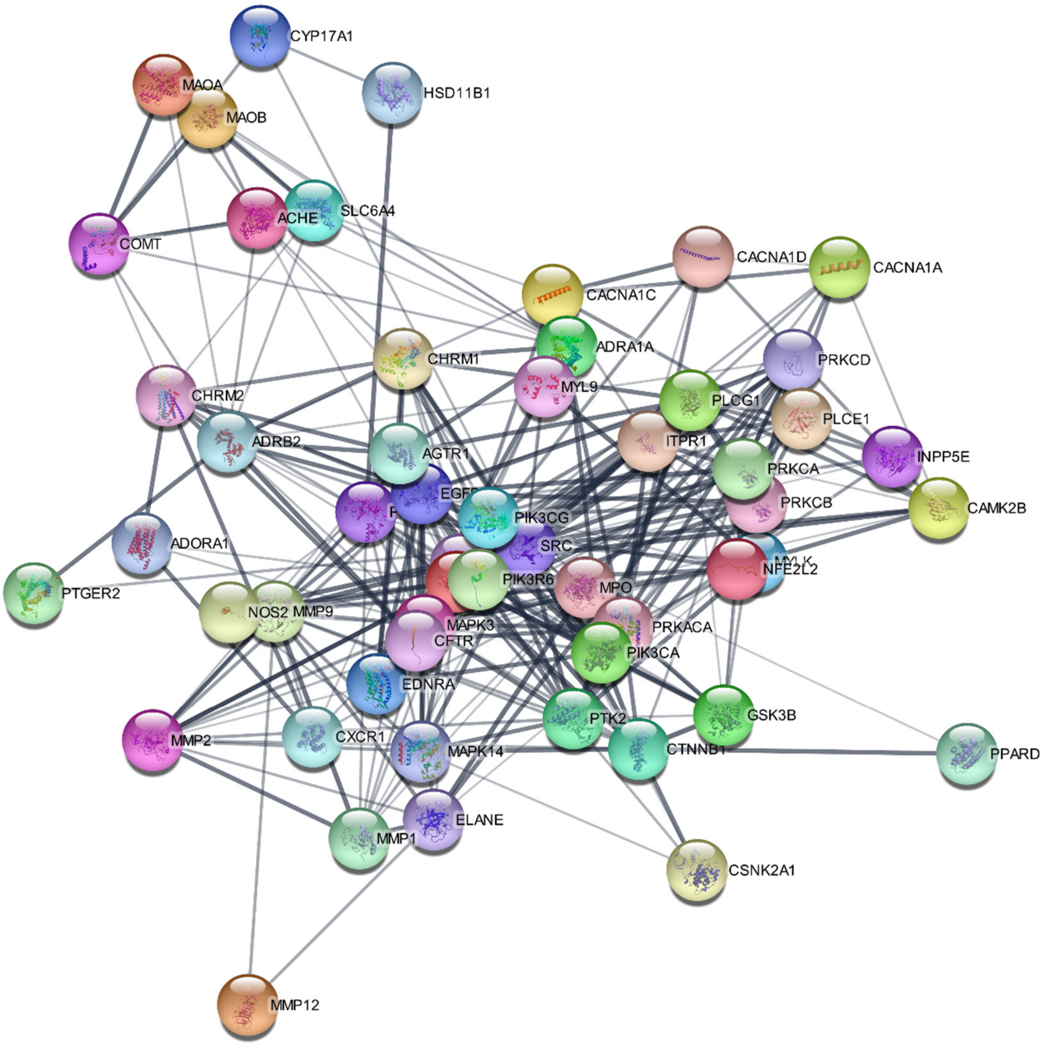

Figure S2. Protein-protein interaction of target genes of respiratory and gastrointestinal disorders.

Table S1. Precision validation of analytical method

Analytes	Theoretical concentration (µg ml $^{-1}$)	Inter-day precision (*n* = 5)		Intra-day precision (*n*=5)	
		Measured concentration (µg ml $^{-1}$)	RSD %	Measured concentration (µg ml $^{-1}$)	RSD %
Stigmasterol	100	99.28± 1.10	1.11	99.45± 1.66	1.67
β-sitosterol	100	99.67± 0.44	0.44	99.36± 0.68	0.68
Scopoletin	100	99.51± 0.96	0.97	99.02± 1.58	1.60
1,4-Dicaffeoylquinic acid	100	99.29± 0.99	0.99	99.47± 1.20	1.20
Luteolin	100	99.69± 0.99	0.99	99.30± 0.39	0.40
Kaempferol	100	99.45± 1.66	1.67	98.57± 1.40	1.42
Ferulic acid	100	99.06± 0.71	0.71	99.05± 1.42	1.44
Epicatechin	100	99.31± 1.32	1.33	98.77± 1.37	1.39
Ellagic acid	100	99.16± 1.27	1.29	99.08± 0.91	0.92
Kaempferol-3-O-glucoside	100	99.48± 0.48	0.48	98.96± 1.27	1.28
Quercetin	100	98.69± 1.72	1.75	99.40± 1.12	1.13
Apigenin	100	99.42± 0.73	0.73	99.42± 0.88	0.89

All values are mean ± S.D. taken as triplicates. All values are mean ± S.D. triplicate analyses for 3 days. Percent coefficient of variation (% RSD); (SD/Mean) ×100.

Table S2. Accuracy validation of analytical method of the *C. sativus* extract through the percent recovery method.

Analytes	Standard additions µg/ml	% Recovery			Mean± SD	RSD%
		Day 1	Day 2	Day 3		
Stigmasterol	50.00	49.99	49.00	49.00	48.50± 1.27	2.62
	100.00	100.00	97.65	99.00	98.93± 0.92	0.93
	200.00	200.00	199.00	197.00	198.22± 1.30	0.66
β-sitosterol	50.00	49.00	49.50	50.60	49.64± 0.70	1.41
	100.00	101.00	98.45	99.55	99.62± 0.99	0.99
	200.00	197.60	197.00	198.10	197.56± 0.55	0.28
Scopoletin	50.00	49.00	49.00	50.10	49.84± 0.88	1.76
	100.00	98.00	99.00	100.10	99.64± 1.18	1.18
	200.00	197.00	197.00	198.10	198.64± 1.84	0.93
1,4-Dicaffeoylquinic acid	50.00	49.00	50.00	51.10	48.92± 1.74	3.56
	100.00	99.00	100.00	101.10	99.62± 1.01	1.02
	200.00	197.00	200.00	201.10	199.84± 1.68	0.84
Luteolin	50.00	51.00	46.70	46.70	47.78± 1.86	3.90
	100.00	100.00	98.45	98.45	98.98± 0.74	0.75
	200.00	200.00	200.00	197.00	198.42± 1.51	0.76
Kaempferol	50.00	49.00	46.70	49.00	48.76± 1.25	2.56
	100.00	98.00	98.45	99.00	98.37± 0.55	0.56
	200.00	197.00	197.00	197.00	197.22± 0.49	0.25
Ferulic acid	50.00	49.00	49.00	50.00	48.62± 1.13	2.32
	100.00	99.00	99.00	100.00	100.22± 1.34	1.34
	200.00	197.00	199.00	200.10	200.64± 2.91	1.45
Epicatechin	50.00	51.00	49.00	50.10	49.84± 0.85	1.71
	100.00	100.00	98.00	99.10	99.24± 0.86	0.86
	200.00	200.00	197.00	198.10	198.84± 1.31	0.66
Ellagic acid	50.00	47.23	47.00	48.10	49.09± 2.32	4.72
	100.00	99.32	101.00	102.10	100.70± 1.07	1.07
	200.00	199.20	203.00	204.10	201.48± 2.04	1.01
Kaempferol-3-O-glucoside	50.00	49.00	49.00	50.10	49.66± 0.72	1.45
	100.00	98.22	99.00	100.10	99.33± 0.83	0.84
	200.00	198.33	199.00	199.00	199.00± 0.56	0.28
Quercetin	50.00	49.00	51.00	49.00	49.89± 0.95	1.91
	100.00	99.00	100.00	97.65	99.10± 0.97	0.98
	200.00	197.67	200.00	199.00	199.11± 0.96	0.48
Apigenin	50.00	49.40	49.56	48.00	49.21± 0.78	1.59
	100.00	99.33	101.00	98.50	99.83± 1.03	1.03
	200.00	199.00	203.00	197.60	200.09± 2.04	1.02

All values are mean ± S.D. taken as triplicates. All values are mean ± S.D. triplicate analyses for 3 days. Percent relative of SD variation (% RSD); (SD/Mean) ×100.

Table S3. Gastrointestinal and respiratory pathogenic genes of the extract.

#	Symbol	Description	Type	Matched Phenotypes	Matched Phenotypes Count	Score	-Log10(p)	Average Disease Causing Likelihood
1	ADRB2	Adrenoceptor Beta 2	Protein	asthma; coughing; wheezing; diarrhea; constipation; "irritable bowel syndrome"	6	80.37	3.93	41
2	SLC6A4	Solute Carrier Family 6 Member 4	Protein	asthma; coughing; wheezing; diarrhea; constipation; "irritable bowel syndrome"	6	56.77	3.29	73
3	CFTR	CF Transmembrane Conductance Regulator	Protein	asthma; coughing; wheezing; diarrhea; constipation; "irritable bowel syndrome"	6	48.87	2.99	17.2
4	MPO	Myeloperoxidase	Protein	asthma; coughing; wheezing; diarrhea; constipation; "irritable bowel syndrome"	6	36.21	2.66	33.8
5	PTGER2	Prostaglandin E Receptor 2	Protein	asthma; coughing; wheezing; diarrhea; constipation	5	34.51	2.71	77.4
6	MYLK	Myosin Light Chain Kinase	Protein	asthma; coughing; wheezing; diarrhea; constipation; "irritable bowel syndrome"	6	32.56	2.62	22.6
7	COMT	Catechol-O-Methyltransferase	Protein	asthma; coughing; wheezing; diarrhea; constipation; "irritable bowel syndrome"	6	32.47	2.61	11.8
8	EGFR	Epidermal Growth Factor Receptor	Protein	asthma; coughing; wheezing; diarrhea; constipation; "irritable bowel syndrome"	6	30.35	2.53	77.6

9	NOS2	Nitric Oxide Synthase 2	Protein	asthma; coughing; wheezing; diarrhea; constipation; "irritable bowel syndrome"	6	28.35	2.44	46.6
10	MMP9	Matrix Metallopeptidase 9	Protein	asthma; coughing; wheezing; diarrhea; constipation; "irritable bowel syndrome"	6	24.8	2.28	20.3
11	ELANE	Elastase, Neutrophil Expressed	Protein	asthma; coughing; wheezing; diarrhea; constipation; "irritable bowel syndrome"	6	24.61	2.27	51.2
12	MMP1	Matrix Metallopeptidase 1	Protein	asthma; coughing; wheezing; diarrhea; constipation	5	23.7	2.34	34.2
13	PPARG	Peroxisome Proliferator Activated Receptor Gamma	Protein	asthma; coughing; wheezing; diarrhea; constipation; "irritable bowel syndrome"	6	21.39	2.21	58.4
14	AKT1	AKT Serine/Threonine Kinase 1	Protein	asthma; coughing; wheezing; diarrhea; constipation; "irritable bowel syndrome"	6	21.07	2.19	90.8
15	PIK3CA	Phosphatidylinositol-4,5-Bisphosphate 3-Kinase Catalytic Subunit Alpha	Protein	asthma; coughing; wheezing; diarrhea; constipation	5	18	2.14	64.4
16	MAPK14	Mitogen-Activated Protein Kinase 14	Protein	asthma; coughing; wheezing; diarrhea; constipation; "irritable bowel syndrome"	6	15.45	1.91	60.3
17	MAPK3	Mitogen-Activated Protein Kinase 3	Protein	asthma; coughing; wheezing; diarrhea; constipation	5	15.07	1.98	84.7
18	CTNNB1	Catenin Beta 1	Protein	asthma; coughing; wheezing; diarrhea; constipation; "irritable bowel syndrome"	6	15.02	1.89	91.9
19	MMP12	Matrix Metallopeptidase 12	Protein	asthma; coughing; wheezing; diarrhea; constipation	5	14.75	1.96	ND

20	MMP2	Matrix Metallopeptidase 2	Protein	asthma; coughing; wheezing; diarrhea; constipation	5	14.69	1.95	71.8
21	AGTR1	Angiotensin II Receptor Type 1	Protein	asthma; coughing; wheezing; diarrhea; constipation	5	14.52	1.94	63.8
22	ADORA1	Adenosine A1 Receptor	Protein	asthma; coughing; wheezing; diarrhea; constipation	5	13.8	1.9	79
23	PIK3CG	Phosphatidylinositol-4,5-Bisphosphate 3-Kinase Catalytic Subunit Gamma	Protein	asthma; coughing; wheezing; diarrhea; constipation	5	12.74	1.81	53.7
24	CXCR1	C-X-C Motif Chemokine Receptor 1	Protein	asthma; coughing; wheezing; diarrhea; constipation	5	12.63	1.8	21.2
25	SRC	SRC Proto-Oncogene, Non-Receptor Tyrosine Kinase	Protein	asthma; coughing; wheezing; diarrhea; constipation	5	11.05	1.71	92
26	CACNA1A	Calcium Voltage-Gated Channel Subunit Alpha1 A	Protein	coughing; diarrhea; constipation; "irritable bowel syndrome"	4	8.07	1.59	51.4
27	PRKCA	Protein Kinase C Alpha	Protein	asthma; coughing; wheezing; diarrhea; constipation	5	8.05	1.52	89.4
28	PRKG1	Protein Kinase CGMP-Dependent 1	Protein	asthma; coughing; wheezing; diarrhea	4	7.63	1.56	66.6
29	HMGCR	3-Hydroxy-3-Methylglutaryl-CoA Reductase	Protein	asthma; coughing; wheezing; diarrhea; constipation; "irritable bowel syndrome"	6	6.7	1.39	83.1
30	ACHE	Acetylcholinesterase (Cartwright Blood Group)	Protein	asthma; coughing; wheezing; diarrhea; constipation	5	6.57	1.43	43.9
31	ACTA2	Actin Alpha 2, Smooth Muscle	Protein	asthma; coughing; wheezing; constipation	4	6.43	1.47	82.6
32	NFE2L2	Nuclear Factor, Erythroid 2 Like 2	Protein	asthma; coughing; wheezing; diarrhea; constipation	5	6.26	1.4	54.7
33	MAOA	Monoamine Oxidase A	Protein	asthma; coughing; diarrhea; constipation	4	6.08	1.45	84.1

34	INPP5E	Inositol Polyphosphate-5-Phosphatase E	Protein	coughing; wheezing; constipation	3	5.77	1.49	51.2
35	EDNRA	Endothelin Receptor Type A	Protein	asthma; coughing; diarrhea; constipation	4	4.65	1.31	84.7
36	PTK2	Protein Tyrosine Kinase 2	Protein	asthma; coughing; wheezing; diarrhea; constipation	5	4.17	1.23	83
37	CHRM2	Cholinergic Receptor Muscarinic 2	Protein	asthma; coughing; constipation	3	2.86	1.19	63.1
38	PRKCB	Protein Kinase C Beta	Protein	asthma; coughing; wheezing; diarrhea; constipation	5	2.82	1.09	87.4
39	PLCG1	Phospholipase C Gamma 1	Protein	asthma; coughing; wheezing; diarrhea; constipation	5	2.71	1.07	65.3
40	MAOB	Monoamine Oxidase B	Protein	asthma; coughing; diarrhea; constipation	4	2.66	1.11	84.5
41	PRKCD	Protein Kinase C Delta	Protein	asthma; coughing; diarrhea; constipation	4	2.51	1.08	81.3
42	CHRM1	Cholinergic Receptor Muscarinic 1	Protein	asthma; coughing; wheezing; constipation	4	2.39	1.07	87.1
43	CACNA1C	Calcium Voltage-Gated Channel Subunit Alpha1 C	Protein	asthma; coughing; diarrhea; constipation	4	2.16	1.03	70.8
44	CYP17A1	Cytochrome P450 Family 17 Subfamily A Member 1	Protein	asthma; coughing; diarrhea; constipation	4	1.85	0.98	90.8
45	CSNK2A1	Casein Kinase 2 Alpha 1	Protein	diarrhea; constipation	2	1.81	1.09	83.5
46	GSK3B	Glycogen Synthase Kinase 3 Beta	Protein	asthma; diarrhea; constipation	3	1.79	1.01	83.2
47	PPARD	Peroxisome Proliferator Activated Receptor Delta	Protein	asthma; diarrhea; constipation; "irritable bowel syndrome"	4	1.47	0.9	93.3
48	PLCE1	Phospholipase C Epsilon 1	Protein	coughing; diarrhea; constipation	3	1.01	0.82	47.3

49	KDM5C	Lysine Demethylase 5C	Protein	asthma; coughing; constipation	3	0.99	0.81	93
50	MYL9	Myosin Light Chain 9	Protein	asthma; diarrhea; constipation	3	0.99	0.81	79.5
51	ADRA1A	Adrenoceptor Alpha 1A	Protein	asthma; coughing	2	0.83	0.82	51
52	CACNA1D	Calcium Voltage-Gated Channel Subunit Alpha1 D	Protein	coughing; constipation	2	0.82	0.82	64.4
53	CAMK2B	Calcium/Calmodulin Dependent Protein Kinase II Beta	Protein	constipation	1	0.81	0.94	50.7
54	ITPR1	Inositol 1,4,5-Trisphosphate Receptor Type 1	Protein	asthma	1	0.38	0.68	73.5
55	HSD11B1	Hydroxysteroid 11-Beta Dehydrogenase 1	Protein	asthma	1	0.29	0.6	87.8
56	CAMK2A	Calcium/Calmodulin Dependent Protein Kinase II Alpha	Protein	constipation	1	0.13	0.34	91.8
57	PRKACA	Protein Kinase CAMP-Activated Catalytic Subunit Alpha	Protein	coughing	1	0.11	0.33	88.8

Table S4. Top 15 GO Biological process of bioactive compounds of an hydroethanolic extract of *C. sativus* for gastrointestinal and respiratory target genes.

ID	Description	pvalue	p.adjust	qvalue	geneID
GO:0050804	modulation of chemical synaptic transmission	0.000000000000089	0.000000000077	0.000000000038	EGFR, ACHE, SLC6A4, CHRM2, PRKCB, GSK3B, SRC, ADORA1, CAMK2B, ADRA1A, ADRB2, CACNA1D, CAMK2A, PLCG1, CACNA1A, PRKACA
GO:0099177	regulation of trans-synaptic signaling	0.000000000000092	0.000000000077	0.000000000038	EGFR, ACHE, SLC6A4, CHRM2, PRKCB, GSK3B, SRC, ADORA1, CAMK2B, ADRA1A, ADRB2, CACNA1D, CAMK2A, PLCG1, CACNA1A, PRKACA
GO:0006874	cellular calcium ion homeostasis	0.000000000000095	0.000000000077	0.000000000038	PTGER2, PRKCB, ADORA1, CXCR1, PIK3CG, AGTR1, CACNA1C, EDNRA, ADRA1A, ELANE, CACNA1D, PLCE1, PLCG1, ITPR1, CACNA1A, PRKACA
GO:0003012	muscle system process	0.000000000000137	0.000000000077	0.000000000038	CHRM2, PTK2, ADORA1, CAMK2B, MYLK, PIK3CG, CACNA1C, EDNRA, ADRA1A, MYL9, ADRB2, CACNA1D, PRKCA, PIK3CA, PLCE1, PRKACA
GO:0055074	calcium ion homeostasis	0.000000000000142	0.000000000077	0.000000000038	PTGER2, PRKCB, ADORA1, CXCR1, PIK3CG, AGTR1, CACNA1C, EDNRA, ADRA1A, ELANE, CACNA1D, PLCE1, PLCG1, ITPR1, CACNA1A, PRKACA
GO:0072503	cellular divalent inorganic cation homeostasis	0.000000000000305	0.000000000138	0.000000000067	PTGER2, PRKCB, ADORA1, CXCR1, PIK3CG, AGTR1, CACNA1C, EDNRA, ADRA1A, ELANE, CACNA1D, PLCE1, PLCG1, ITPR1, CACNA1A, PRKACA
GO:0051480	regulation of cytosolic calcium ion concentration	0.000000000000959	0.000000000372	0.000000000182	PTGER2, ADORA1, CXCR1, PIK3CG, AGTR1, CACNA1C, EDNRA, ADRA1A, CACNA1D, PLCE1, PLCG1, ITPR1, CACNA1A, PRKACA
GO:0018105	peptidyl-serine phosphorylation	0.000000000003001	0.000000001019	0.000000000498	EGFR, PRKCD, PRKCB, GSK3B, SRC, CSNK2A1, AKT1, MAPK3, MAPK14, PRKCA, PIK3CA, CAMK2A, PRKACA
GO:1901653	cellular response to peptide	0.000000000004155	0.000000001254	0.000000000613	NFE2L2, PPARG, PRKCD, PRKCB, PTK2, GSK3B, SRC, AKT1, AGTR1, ADRB2, PIK3CA, CAMK2A, CACNA1A, PRKACA
GO:0007204	positive regulation of cytosolic calcium ion concentration	0.000000000004844	0.000000001316	0.000000000643	PTGER2, CXCR1, PIK3CG, AGTR1, CACNA1C, EDNRA, ADRA1A, CACNA1D, PLCE1, PLCG1, ITPR1, CACNA1A, PRKACA
GO:0090257	regulation of muscle system process	0.000000000006234	0.000000001540	0.000000000752	CHRM2, PTK2, ADORA1, CAMK2B, PIK3CG, CACNA1C, ADRA1A, MYL9, ADRB2, PRKCA, PLCE1, PRKACA
GO:0018209	peptidyl-serine modification	0.000000000007390	0.000000001673	0.000000000817	EGFR, PRKCD, PRKCB, GSK3B, SRC, CSNK2A1, AKT1, MAPK3, MAPK14, PRKCA, PIK3CA, CAMK2A, PRKACA
GO:0038127	ERBB signaling pathway	0.000000000009425	0.000000001970	0.000000000962	MMP9, EGFR, PTK2, SRC, ADORA1, AKT1, PRKCA, PIK3CA, PLCE1, PLCG1
GO:0006936	muscle contraction	0.000000000021020	0.000000004025	0.000000001966	CHRM2, ADORA1, MYLK, PIK3CG, CACNA1C, EDNRA, ADRA1A, MYL9, ADRB2, CACNA1D, PIK3CA, PLCE1, PRKACA

GO:0030168	platelet activation	0.000000000022218	0.000000004025	0.000000001966	PIK3R6, PRKCD, PRKCB, SRC, PIK3CG, MYL9, MAPK3, PRKCA, PIK3CA, ITPR1

Table S5: Top 15 KEGG pathway of bioactive compounds of a hydroethanolic extract of *C. sativus* for gastrointestinal and respiratory target genes.

ID	Description	pvalue	p.adjust	qvalue	geneID
hsa04725	Cholinergic synapse	0.00000000000000	0.00000000000000	0.00000000000000	CHRM1, PIK3R6, ACHE, CHRM2, PRKCB, CAMK2B, AKT1, PIK3CG, CACNA1C, CACNA1D, MAPK3, PRKCA, PIK3CA, CAMK2A, ITPR1, CACNA1A, PRKACA
hsa04020	Calcium signaling pathway	0.00000000000000	0.00000000000000	0.00000000000000	CHRM1, EGFR, NOS2, CHRM2, PRKCB, CAMK2B, MYLK, AGTR1, CACNA1C, EDNRA, ADRA1A, ADRB2, CACNA1D, PRKCA, CAMK2A, PLCE1, PLCG1, ITPR1, CACNA1A, PRKACA
hsa05205	Proteoglycans in cancer	0.00000000000000	0.00000000000002	0.00000000000000	MMP9, MMP2, EGFR, CTNNB1, PRKCB, PTK2, SRC, CAMK2B, AKT1, MAPK3, MAPK14, PRKCA, PIK3CA, CAMK2A, PLCE1, PLCG1, ITPR1, PRKACA
hsa04912	GnRH signaling pathway	0.00000000000000	0.00000000000002	0.00000000000000	MMP2, EGFR, PRKCD, PRKCB, SRC, CAMK2B, CACNA1C, CACNA1D, MAPK3, MAPK14, PRKCA, CAMK2A, ITPR1, PRKACA
hsa04728	Dopaminergic synapse	0.00000000000000	0.00000000000010	0.00000000000002	MAOB, MAOA, PRKCB, GSK3B, COMT, CAMK2B, AKT1, CACNA1C, CACNA1D, MAPK14, PRKCA, CAMK2A, ITPR1, CACNA1A, PRKACA
hsa04024	cAMP signaling pathway	0.00000000000001	0.00000000000044	0.00000000000009	CHRM1, CHRM2, PTGER2, ADORA1, CAMK2B, AKT1, CFTR, CACNA1C, EDNRA, MYL9, ADRB2, CACNA1D, MAPK3, PIK3CA, CAMK2A, PLCE1, PRKACA
hsa04921	Oxytocin signaling pathway	0.00000000000003	0.00000000000076	0.00000000000016	EGFR, PIK3R6, PRKCB, SRC, CAMK2B, MYLK, PIK3CG, CACNA1C, MYL9, CACNA1D, MAPK3, PRKCA, CAMK2A, ITPR1, PRKACA
hsa04012	ErbB signaling pathway	0.00000000000015	0.00000000000387	0.00000000000081	EGFR, PRKCB, PTK2, GSK3B, SRC, CAMK2B, AKT1, MAPK3, PRKCA, PIK3CA, CAMK2A, PLCG1
hsa05417	Lipid and atherosclerosis	0.00000000000021	0.00000000000494	0.00000000000103	MMP9, MMP1, NFE2L2, PPARG, PTK2, GSK3B, SRC, CAMK2B, AKT1, MAPK3, MAPK14, PRKCA, PIK3CA, CAMK2A, PLCG1, ITPR1
hsa04261	Adrenergic signaling in cardiomyocytes	0.00000000000039	0.00000000000731	0.00000000000152	PIK3R6, CAMK2B, AKT1, PIK3CG, AGTR1, CACNA1C, ADRA1A, ADRB2, CACNA1D, MAPK3, MAPK14, PRKCA, CAMK2A, PRKACA
hsa04935	Growth hormone synthesis, secretion and action	0.00000000000039	0.00000000000731	0.00000000000152	PRKCB, PTK2, GSK3B, AKT1, CACNA1C, CACNA1D, MAPK3, MAPK14, PRKCA, PIK3CA, PLCG1, ITPR1, PRKACA
hsa04750	Inflammatory mediator regulation of TRP channels	0.00000000000087	0.00000000001499	0.00000000000313	PTGER2, PRKCD, PRKCB, SRC, CAMK2B, MAPK14, PRKCA, PIK3CA, CAMK2A, PLCG1, ITPR1, PRKACA
hsa04270	Vascular smooth muscle contraction	0.00000000000166	0.00000000002547	0.00000000000531	PRKCD, PRKCB, MYLK, AGTR1, CACNA1C, EDNRA, ADRA1A, MYL9, CACNA1D, MAPK3, PRKCA, ITPR1, PRKACA
hsa04022	cGMP-PKG signaling pathway	0.00000000000172	0.00000000002547	0.00000000000531	PIK3R6, ADORA1, AKT1, MYLK, PIK3CG, AGTR1, CACNA1C, EDNRA, ADRA1A, MYL9, ADRB2, CACNA1D, MAPK3, ITPR1

hsa04926	Relaxin signaling pathway	0.000000 00002417	0.000000 00033353	0.000000 00006954	MMP9, MMP1, MMP2, EGFR, NOS2, SRC, AKT1, MAPK3, MAPK14, PRKCA, PIK3CA, PRKACA

Table S6. Network analysis of pathogenic target genes interaction with phytoconstituents.

Name	Betweenness Centrality	Closeness Centrality	Degree	Topological Coefficient	Type
Kaempferol	0.30	0.50	27	0.30	Compounds
Quercetin	0.15	0.46	21	0.37	Compounds
Apigenin	0.09	0.43	17	0.42	Compounds
Luteolin	0.06	0.43	17	0.45	Compounds
Sitosterol	0.18	0.42	14	0.20	Compounds
Kaempferol 3-O-glucoside	0.11	0.41	13	0.31	Compounds
Ellagic acid	0.06	0.41	12	0.41	Compounds
Stigmasterol	0.09	0.40	11	0.22	Compounds
Ferulic acid	0.12	0.40	10	0.30	Compounds
Scopoletin	0.07	0.39	10	0.42	Compounds
ACHE	0.19	0.50	9	0.30	Genes
1,4-Dicaffeoylquinic acid	0.07	0.38	8	0.33	Compounds
MMP9	0.05	0.45	7	0.34	Genes
MMP2	0.05	0.45	7	0.34	Genes
EGFR	0.04	0.43	7	0.37	Genes
MAOA	0.04	0.43	7	0.37	Genes
PTK2	0.01	0.40	6	0.45	Genes
GSK3B	0.01	0.40	6	0.45	Genes
SRC	0.01	0.40	6	0.45	Genes
ADORA1	0.01	0.39	5	0.50	Genes
CSNK2A1	0.01	0.38	5	0.52	Genes
MMP12	0.02	0.40	5	0.46	Genes
CACNA1D	0.05	0.42	5	0.37	Genes
PRKCD	0.02	0.35	4	0.40	Genes
PRKCB	0.02	0.35	4	0.40	Genes
MPO	0.00	0.37	4	0.61	Genes
CXCR1	0.00	0.37	4	0.61	Genes
CAMK2B	0.00	0.37	4	0.61	Genes
AKT1	0.00	0.37	4	0.55	Genes
HSD11B1	0.01	0.32	3	0.51	Genes
NOS2	0.01	0.32	3	0.51	Genes
MYLK	0.01	0.38	3	0.55	Genes
CFTR	0.00	0.35	3	0.69	Genes
MAOB	0.00	0.31	2	0.56	Genes
MMP1	0.00	0.30	2	0.57	Genes
CHRM1	0.00	0.32	2	0.66	Genes
PIK3R6	0.00	0.31	2	0.50	Genes
CYP17A1	0.00	0.30	2	0.88	Genes
SLC6A4	0.00	0.30	2	0.88	Genes

CHRM2	0.00	0.30	2	0.88	Genes
PTGER2	0.00	0.30	2	0.88	Genes
PPARD	0.00	0.30	2	0.88	Genes
PPARG	0.00	0.30	2	0.88	Genes
PIK3CG	0.00	0.34	2	0.59	Genes
ADRA1A	0.00	0.35	2	0.66	Genes
PRKCA	0.00	0.36	2	0.61	Genes
CAMK2A	0.00	0.36	2	0.61	Genes
ITPR1	0.00	0.32	2	0.66	Genes
CACNA1A	0.00	0.32	2	0.82	Genes
PRKACA	0.00	0.32	2	0.55	Genes
NFE2L2	0.00	0.28	1	0.00	Genes
CTNNB1	0.00	0.28	1	0.00	Genes
COMT	0.00	0.28	1	0.00	Genes
AGTR1	0.00	0.32	1	0.00	Genes
CACNA1C	0.00	0.34	1	0.00	Genes
EDNRA	0.00	0.34	1	0.00	Genes
MYL9	0.00	0.34	1	0.00	Genes
ADRB2	0.00	0.34	1	0.00	Genes
INPP5E	0.00	0.30	1	0.00	Genes
ELANE	0.00	0.27	1	0.00	Genes
MAPK3	0.00	0.29	1	0.00	Genes
MAPK14	0.00	0.29	1	0.00	Genes
PIK3CA	0.00	0.29	1	0.00	Genes
PLCE1	0.00	0.34	1	0.00	Genes
PLCG1	0.00	0.34	1	0.00	Genes

Table S7. Network analysis of pathogenic target genes ginteraction with phytoconstituents and GO biological process (BP).

Name	Betweenness Centrality	Closeness Centrality	Degree	Topological Coefficient	Type
Kaempferol	0.09	0.52	123	0.33	Compounds
ADORA1	0.03	0.48	100	0.41	Genes
CACNA1D	0.04	0.50	90	0.36	Genes
SRC	0.03	0.48	84	0.34	Genes
Quercetin	0.05	0.47	80	0.34	Compounds
Kaempferol 3-O-glucoside	0.03	0.45	77	0.37	Compounds
PRKCB	0.05	0.50	72	0.31	Genes
EGFR	0.04	0.47	70	0.33	Genes
Ellagic acid	0.03	0.44	62	0.38	Compounds
Regulation of trans-synaptic signaling	0.04	0.47	60	0.38	BP
GSK3B	0.02	0.47	60	0.35	Genes
Modulation of chemical synaptic transmission	0.04	0.47	60	0.38	BP
Luteolin	0.02	0.45	58	0.38	Compounds
Apigenin	0.02	0.44	53	0.37	Compounds
PRKACA	0.05	0.52	52	0.34	Genes
PTK2	0.02	0.44	48	0.36	Genes
Peptidyl-serine modification	0.04	0.45	45	0.32	BP
Peptidyl-serine phosphorylation	0.04	0.45	45	0.32	BP
Sitosterol	0.03	0.42	42	0.28	Compounds
Cellular response to peptide	0.07	0.46	42	0.31	BP
ERBB signaling pathway	0.02	0.42	40	0.40	BP
CXCR1	0.01	0.42	40	0.49	Genes
ADRA1A	0.02	0.46	40	0.43	Genes
Muscle system process	0.04	0.46	39	0.37	BP
Cellular divalent inorganic cation homeostasis	0.04	0.47	36	0.38	BP
Calcium ion homeostasis	0.04	0.47	36	0.38	BP
PIK3CG	0.02	0.46	36	0.38	Genes
Cellular calcium ion homeostasis	0.04	0.47	36	0.38	BP
ACHE	0.03	0.47	36	0.32	Genes
AKT1	0.01	0.44	32	0.38	Genes
PRKCD	0.02	0.42	32	0.31	Genes
CACNA1A	0.02	0.44	32	0.42	Genes
CAMK2B	0.01	0.42	32	0.47	Genes
Cegulation of cytosolic calcium ion concentration	0.02	0.45	31	0.40	BP
Scopoletin	0.00	0.40	29	0.43	Compounds

Regulation of muscle system process	0.02	0.44	29	0.38	BP
Stigmasterol	0.01	0.41	28	0.29	Compounds
Muscle contraction	0.02	0.44	27	0.40	BP
Positive regulation of cytosolic calcium ion concentration	0.02	0.44	26	0.39	BP
Platelet activation	0.03	0.43	25	0.30	BP
PRKCA	0.01	0.42	24	0.40	Genes
ITPR1	0.01	0.42	24	0.40	Genes
CSNK2A1	0.01	0.42	20	0.41	Genes
PTGER2	0.01	0.37	20	0.55	Genes
CAMK2A	0.01	0.43	20	0.42	Genes
CHRM2	0.01	0.41	20	0.41	Genes
1,4-Dicaffeoylquinic acid	0.01	0.38	18	0.33	Compounds
PLCE1	0.01	0.43	18	0.45	Genes
CACNA1C	0.01	0.43	16	0.46	Genes
PLCG1	0.01	0.43	16	0.45	Genes
MMP9	0.02	0.43	14	0.32	Genes
PIK3CA	0.01	0.44	14	0.34	Genes
EDNRA	0.01	0.42	14	0.48	Genes
MYLK	0.00	0.40	12	0.49	Genes
AGTR1	0.01	0.42	12	0.45	Genes
ADRB2	0.01	0.42	12	0.46	Genes
MYL9	0.00	0.40	8	0.45	Genes
SLC6A4	0.00	0.35	8	0.56	Genes
Ferulic acid	0.00	0.35	7	0.45	Compounds
MAPK3	0.00	0.36	6	0.48	Genes
ELANE	0.00	0.34	6	0.68	Genes
PIK3R6	0.00	0.35	4	0.38	Genes
PPARG	0.00	0.35	4	0.47	Genes
MAPK14	0.00	0.34	4	0.61	Genes
NFE2L2	0.00	0.33	2	0.50	Genes

Table S8. Network analysis of pathogenic target genes interaction with phytoconstituents and KEGG pathway.

Name	Betweenness Centrality	Closeness Centrality	Degree	Topological Coefficient	Type
Kaempferol	0.09	0.51	126	0.34	Compounds
CACNA1D	0.03	0.49	100	0.35	Genes
CAMK2B	0.03	0.51	88	0.34	Genes
Kaempferol 3-O-glucoside	0.02	0.44	84	0.38	Compounds
SRC	0.02	0.49	84	0.32	Genes
EGFR	0.03	0.49	84	0.32	Genes
Quercetin	0.04	0.47	81	0.34	Compounds
AKT1	0.03	0.50	80	0.34	Genes
PRKCB	0.04	0.49	80	0.30	Genes
Luteolin	0.02	0.44	63	0.39	Compounds
Ellagic acid	0.02	0.43	63	0.39	Compounds
Proteoglycans in cancer	0.05	0.47	59	0.40	KEGG
Sitosterol	0.02	0.43	52	0.30	Compounds
PRKCA	0.03	0.51	52	0.33	Genes
Lipid and atherosclerosis	0.06	0.46	48	0.36	KEGG
GSK3B	0.01	0.45	48	0.35	Genes
GnRH signaling pathway	0.02	0.45	48	0.46	KEGG
PTK2	0.01	0.45	48	0.35	Genes
Calcium signaling pathway	0.07	0.49	48	0.32	KEGG
PRKACA	0.03	0.49	48	0.33	Genes
Cholinergic synapse	0.04	0.46	47	0.41	KEGG
Apigenin	0.02	0.43	46	0.36	Compounds
Dopaminergic synapse	0.05	0.45	45	0.39	KEGG
ErbB signaling pathway	0.01	0.44	44	0.45	KEGG
Oxytocin signaling pathway	0.03	0.45	44	0.42	KEGG
ITPR1	0.03	0.49	44	0.33	Genes
CAMK2A	0.02	0.50	44	0.35	Genes
Relaxin signaling pathway	0.02	0.44	43	0.39	KEGG
MMP2	0.02	0.45	42	0.33	Genes
MMP9	0.02	0.46	42	0.32	Genes
Scopoletin	0.02	0.42	39	0.33	Compounds
cAMP signaling pathway	0.05	0.45	38	0.34	KEGG
Growth hormone synthesis, secretion and action	0.02	0.44	36	0.45	KEGG

cGMP-PKG signaling pathway	0.02	0.44	31	0.32	KEGG
Inflammatory mediator regulation of TRP channels	0.02	0.43	31	0.42	KEGG
Adrenergic signaling in cardiomyocytes	0.02	0.43	30	0.43	KEGG
Vascular smooth muscle contraction	0.02	0.43	29	0.39	KEGG
1,4-Dicaffeoylquinic acid	0.01	0.40	25	0.33	Compounds
PRKCD	0.01	0.39	24	0.32	Genes
MYLK	0.01	0.42	24	0.44	Genes
MAPK3	0.03	0.48	24	0.33	Genes
Stigmasterol	0.01	0.40	22	0.25	Compounds
Ferulic acid	0.03	0.39	20	0.30	Compounds
ADORA1	0.01	0.42	20	0.42	Genes
CACNA1C	0.01	0.46	20	0.38	Genes
ACHE	0.02	0.46	18	0.31	Genes
MAPK14	0.01	0.44	16	0.35	Genes
ADRA1A	0.00	0.41	16	0.42	Genes
PIK3CA	0.01	0.44	16	0.35	Genes
PIK3CG	0.00	0.40	16	0.44	Genes
PIK3R6	0.01	0.40	16	0.36	Genes
MAOA	0.01	0.43	14	0.34	Genes
PLCG1	0.01	0.44	12	0.39	Genes
NOS2	0.01	0.41	12	0.30	Genes
CACNA1A	0.00	0.41	12	0.42	Genes
CHRM2	0.01	0.39	12	0.41	Genes
CHRM1	0.00	0.42	12	0.41	Genes
MMP1	0.00	0.36	8	0.39	Genes
MYL9	0.00	0.40	8	0.45	Genes
PTGER2	0.00	0.36	8	0.40	Genes
ADRB2	0.00	0.41	8	0.46	Genes
EDNRA	0.00	0.41	8	0.46	Genes
AGTR1	0.00	0.40	8	0.43	Genes
CFTR	0.00	0.38	6	0.53	Genes
PLCE1	0.00	0.40	6	0.53	Genes
PPARG	0.00	0.35	4	0.45	Genes
MAOB	0.00	0.36	4	0.41	Genes
NFE2L2	0.00	0.34	2	0.55	Genes
COMT	0.00	0.33	2	0.61	Genes
CTNNB1	0.00	0.34	2	0.57	Genes
